# Phytochemical and Geographical Comparison of *Derris scandens* (Roxb.) Benth. Leaves and Stems and Their Nitric Oxide Production Inhibitory Activity

**DOI:** 10.1155/sci5/2028051

**Published:** 2026-05-29

**Authors:** Benyatip Buajan, Mudtorlep Nisoa, Fonthip Makkliang, Atthaphon Konyanee, Waraporn Putalun, Geoffrey A. Cordell, Rawiwan Charoensup, Gorawit Yusakul

**Affiliations:** ^1^ School of Pharmacy, Walailak University, Nakhon Si Thammarat, 80160, Thailand, wu.ac.th; ^2^ School of Public Health, Walailak University, Nakhon Si Thammarat, 80160, Thailand, wu.ac.th; ^3^ Futuristic Science Research Center, School of Science, Walailak University, Nakhon Si Thammarat, 80160, Thailand, wu.ac.th; ^4^ Functional Materials and Nanotechnology Center of Excellence, Walailak University, Nakhon Si Thammarat, 80160, Thailand, wu.ac.th; ^5^ Hub of Knowledge in Microwave Heating and Applications, Walailak University, Nakhon Si Thammarat, 80160, Thailand, wu.ac.th; ^6^ School of Languages and General Education, Walailak University, Nakhon Si Thammarat, 80160, Thailand, wu.ac.th; ^7^ School of Medicine, Walailak University, Nakhon Si Thammarat, 80160, Thailand, wu.ac.th; ^8^ Research Center in Tropical Pathobiology, Walailak University, Nakhon Si Thammarat, 80160, Thailand, wu.ac.th; ^9^ Faculty of Pharmaceutical Sciences, Khon Kaen University, Khon Kaen, 40002, Thailand, kku.ac.th; ^10^ Natural Products Inc., Evanston, Illinois, 60201, USA, npisoy.com; ^11^ Department of Pharmaceutics, College of Pharmacy, University of Florida, Gainesville, Florida, 32610, USA, ufl.edu; ^12^ Medicinal Plants Innovation Center, Mae Fah Luang University, Chiang Rai, 57100, Thailand, mfu.ac.th; ^13^ School of Integrative Medicine, Mae Fah Luang University, Chiang Rai, 57100, Thailand, mfu.ac.th; ^14^ Department of Pharmaceutical Chemistry and Pharmacognosy, Faculty of Pharmaceutical Sciences, Naresuan University, Phitsanulok, 65000, Thailand, nu.ac.th; ^15^ Research and Innovation Cluster for Natural Health Products, Naresuan University, Phitsanulok, 65000, Thailand, nu.ac.th

**Keywords:** anti-inflammatory activity, *Derris scandens*, isoflavones, leaves, stems

## Abstract

*Derris scandens* (Roxb.) Benth. (Fabaceae) is a medicinal plant with anti‐inflammatory properties. Its stem extract is approved in Thailand for treating musculoskeletal pain. However, sustainability concerns necessitate exploring alternative sources such as leaves. This study aimed to compare the phytochemical profiles and anti‐inflammatory activity of *D. scandens* leaves and stems collected from different geographical locations. High‐performance liquid chromatography with diode‐array detection (HPLC–DAD) was employed to quantify isoflavones in extracts prepared using various ethanol concentrations. Anti‐inflammatory activity was assessed through nitric oxide (NO) secretion inhibition in lipopolysaccharide (LPS)‐induced RAW 264.7 macrophage cells. Geographical location influenced metabolite profiles and biological activity. Leaves contained isoangustone A (up to 26.6 μg/mg) and lupalbigenin (up to 0.98 μg/mg), while stems exhibited higher lupalbigenin levels (up to 296 μg/mg) and additional metabolites, including derrisisoflavone A and 6,8‐diprenylgenistein. At 25 μg/mL, NO inhibition by derrisisoflavone A (34.3%) and 6,8‐diprenylgenistein (23.3%) was higher than that of leaf‐derived compounds such as derrubone (31.0%) and isoangustone A (18.0%). Ethanolic extracts (50 μg/mL) of the stems demonstrated greater NO inhibition (9.44%–59.6%) compared to the leaves (6.59%–39.2%). While *D. scandens* leaves offer an alternative phytochemical resource, their distinct chemical and biological profiles limit their substitution for the stems as a source of anti‐inflammatory metabolites. The findings support the regulatory approval of the stems as an essential Thai medicine. Molecular docking revealed that lupalbigenin showed the strongest binding to COX‐1, COX‐2, and 5‐LOX, supporting its potential as a dual COX/LOX inhibitor for anti‐inflammatory drug development.

## 1. Introduction

**FIGURE 1:**
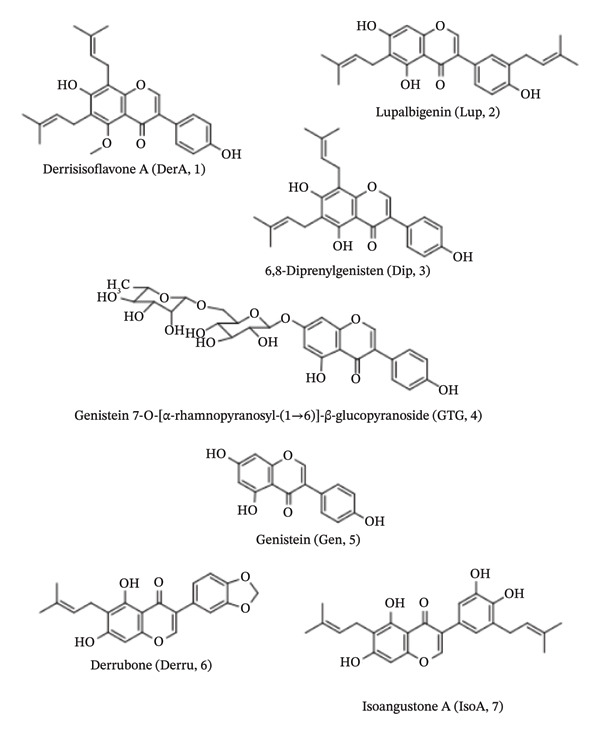
Structures of selected *D. scandens* leaf and stem constituents, including derrisisoflavone A (DerA, **1**), lupalbigenin (Lup, **2**), 6,8‐diprenylgenistein (Dip, **3**), genistein‐7‐*O*‐[2]‐β‐glucopyranoside (GTG, **4**), genistein (Gen, **5**), derrubone (Derru, **6**), and isoangustone A (IsoA, **7**).

The ethanolic extract of the stem of *Derris scandens* (Roxb.) Benth. (Fabaceae) is included in the National List of Essential Medicines of Thailand and is indicated for the treatment of musculoskeletal pain [1]. An increased need and unsustainable harvesting of the stems are leading to shortages of this medicinal plant and to concerns regarding accessibility (affordability and availability) for the patient. Liquid chromatography–electrospray ionization–quadrupole time‐of‐flight mass spectrometry/mass spectrometric (LC–ESI–QTOF–MS/MS) examination of a 95% ethanol stem extract identified several bioactive isoflavones, including derrisisoflavone A (DerA, **1**, Figure 1), lupalbigenin (Lup, **2**, Figure 1), 6,8‐diprenylgenistein (Dip, **3**, Figure 1), genistein‐7‐*O*‐[2]‐β‐glucopyranoside (GTG, **4**, Figure 1), and genistein (Gen, **5**, Figure 1) [2–4]. Some of these compounds, particularly prenylated isoflavones such as Lup (**2**), have demonstrated anti‐inflammatory activity in vitro [4]. Similarly, the leaf extract has been reported to contain structurally related isoflavones, including derrubone (Derru, **6**, Figure 1), Lup (**2**), and isoangustone A (IsoA, **7**, Figure 1) [5], suggesting potential overlap in bioactive constituents between the two plant parts. Flavonoids identified from *D. scandens* leaves include ovaliflavanone and lupinifolin [6]. In a carrageenan‐induced rat paw edema assay, the leaf extract reduced paw thickness by 49.23% at 400 mg/kg, which was comparable to the anti‐inflammatory effect of indomethacin (50% inhibition), while the root extract showed lower activity (37.26% inhibition) [6]. These findings indicate that *D. scandens* leaves possess substantial anti‐inflammatory potential and may serve as a promising alternative source of bioactive compounds.

The metabolite profiles of *D. scandens* leaves and stems each include prenylated isoflavones (Figure 1), suggesting that a preparation of the leaves might serve as an alternative anti‐inflammatory ingredient. However, so far, only Lup (**2**) is recognized as a constituent of the stem. The leaves comprise additional metabolites, including warangalone, Derru (**6**), and glyurallin B, which will alter the bioactivity profile. It remained, therefore, to characterize the anti‐inflammatory metabolites present in the leaves and compare their efficacy with the stem extracts in inflammatory assays. In this way, the *D. scandens* leaf material was evaluated as a sustainable source of anti‐inflammatory metabolites. However, comparative studies of *D. scandens* stems and leaves across regions are scarce, and no validated workflow links isoflavone profiles with bioactivity. This gap limits standardization and sustainable use of alternative plant parts.

Therefore, we hypothesized that *D. scandens* leaves may produce anti‐inflammatory metabolites and could serve as a sustainable and alternative source of stem material. This study aimed to analyze and compare the metabolites of *D. scandens* leaf and stem materials and to evaluate their anti‐inflammatory activity. The findings are expected to provide comparative evidence for future phytopharmaceutical development and for establishing standardized protocols for *D. scandens*.

## 2. Materials and Methods

### 2.1. Chemicals

GTG (**4**, 99% purity) and DerA (**1**, 93% purity) were isolated from *D. scandens* stems and identified using NMR spectroscopy [2, 4, 7]. Dip (**3**, 98% purity) was acquired from Wuhan ChemFaces Biochemical Co., Ltd. (Wuhan, Hubei Province, China), and the authenticity was verified by LC‐ESI‐QTOF‐MS/MS [8]. Gen (**5**, 99.3% purity) was obtained from LKT Laboratories, Inc. (St. Paul, MN, USA). Lup (**2**, 95% purity) was acquired as described in a previous study [9]. Derru (**6**, 93% purity) and IsoA (**7**, 95% purity) were isolated from the leaves of *D. scandens* as described in the Supporting Materials (Figures S1–S2 and Tables S1–S2). Silica gel (high‐purity grade, 60 Å, 70–230 mesh) was obtained from Merck KGaA (St. Louis, MO, USA), and Cosmosil 75C18‐OPN was obtained from Nacalai Tesque, Inc. (Nakagyo‐ku, Kyoto, Japan). Hexane, chloroform, ethyl acetate, acetonitrile, acetic acid (99.8%), absolute ethanol (EtOH), methanol, and deionized water were supplied by RCI Labscan Ltd. (Bangkok, Thailand). Trifluoroacetic acid (99.8%) was obtained from Merck KGaA (Darmstadt, Germany).

### 2.2. Plant Materials

Fresh leaves of *D. scandens* were obtained from three distinct locations in Thailand during May 2023, including the Tha Sala District (Nakhon Si Thammarat Province, **Lf1**), the Si Mueang Mai District (Ubon Ratchathani Province, **Lf2**), and the Wang Saphung District (Loei Province, **Lf3**). Four sources of *D. scandens* stems were collected in June 2023, including the Tha Sala District (Nakhon Si Thammarat Province, **St1**), the Si Mueang Mai District (Ubon Ratchathani Province, **St2** and **St3**), and the Pho Sai District (Ubon Ratchathani Province, **St4**).

The samples were dried at 50°C for 48 h, followed by powdering. The identification of *D. scandens* at the Tha Sala District location was verified by the Forest and Plant Conservation Research Office, Department of National Parks, Wildlife and Plant Conservation, Bangkok, Thailand. Other plant materials were authenticated by Dr. G. Yusakul, Faculty of Pharmaceutical Sciences, Naresuan University, Thailand. Voucher specimens were deposited in the herbarium of The Center for Scientific and Technological Equipment, Walailak University, Thailand.

### 2.3. High‐Performance Liquid Chromatography With Diode‐Array Detection (HPLC–DAD) Analytical Methods for *D. scandens* Extracts

The analytical methods for the metabolites in the extracts were developed in our previous study [10] and comprised three distinct HPLC systems: HPLC System 1 for Derru (**6**), IsoA (**7**), and Lup (**2**) in the leaf extract; HPLC System 2 for GTG (**4**) in the stem extract; and HPLC System 3 for Gen (**5**), DerA (**1**), Dip (**3**), and Lup (**2**) in the stem extract. The HPLC systems utilized a Thermo Scientific Dionex Ultimate 3000 system (Thermo Fisher Scientific, Fremont, CA, USA) with a VWD‐3400 RS detector, WPS‐3000SL autosampler, LPG‐34003D solvent delivery pump, and TCC‐3000SD column compartments (Thermo Fisher Scientific, Fremont, CA, USA).

HPLC System 1 employed a reversed‐phase column (Zorbax Eclipse Plus C8, 4.6 × 250 mm, 5‐μm particle size, Santa Clara, CA, USA) with the column temperature maintained at 35 ± 2°C. The gradient mobile phase consisted of two solvents: (A) 0.5% v/v acetic acid in water and (B) 80% v/v acetonitrile in 0.5% aqueous acetic acid. The proportion of Solvent B to Solvent A was changed according to the following gradient profile: 60% B (0–10 min), 60%–70% (10–50 min), 70%–60% (50–55 min), and hold at 60% A (55–60 min). The mobile phase flow rate was maintained at 1 mL/min throughout the analysis. Compound detection was conducted at 260 nm for Derru (**6**) and IsoA (**7**) and at 268 nm for Lup (**2**).

HPLC System 2 utilized a reverse‐phase column (octadecylsilane, C18, VertiSep UPS, 4.6 × 250 mm, particle size of 5 μm; Vertical Chromatography Co., Ltd., Nonthaburi, Thailand), with the column temperature maintained at 35 ± 2°C. The gradient mobile phase consisted of two solvents: (A) 0.02% trifluoroacetic acid in water, v/v, and (B) 80% acetonitrile in 0.02% aqueous trifluoroacetic acid. The proportions of Solvent B to Solvent A were altered according to the following gradient profile: 15%–70% (0–10 min), 70%–73.5% (10–15 min), 73.5%–15% (15–16 min), and holding at 15% (16–18 min). The flow rate of the mobile phase was 1 mL/min. Detection of GTG (**4**) was conducted at 260 nm.

HPLC System 3 utilized a reverse‐phase column (Zorbax Eclipse Plus C8, 4.6 × 250 mm, 5 μm, Santa Clara, CA, USA), with the column temperature maintained at 35 ± 2°C. The gradient mobile phase comprised two solvents: (A) 0.5% v/v acetic acid in water and (B) 80% v/v acetonitrile in 0.5% aqueous acetic acid. The proportion of Solvent B to Solvent A was altered according to the following gradient profile: 55% (0–10 min), 55%–70% (10–60 min), 70% (60–62 min), 70%–55% (62–65 min), and hold at 55% (65–70 min). The mobile phase flow rate was 1 mL/min. Detection of the compounds was performed at 260 nm for Gen (**5**) and DerA (**1**) and at 268 nm for Lup (**2**) and Dip (**3**).

Analytical performance was validated through the determination of the limit of detection (LOD) and limit of quantification (LOQ) values, as reported previously [10]. These values were calculated utilizing the standard deviation of the signal (*σ*) and the slope of the standard curve (s): LOD, 3.3*σ*/s, and LOQ, 10*σ*/s. Precision analysis of the standard compounds was conducted within the concentration range of the calibration curve and was expressed as the coefficient of variation (%CV), derived from the repeated analysis of the standards (*n* = 3).

In the recovery experiment, different ethanol strengths were selected based on the target compound polarity. For instance, 95% ethanol was used to extract the less polar prenylated isoflavonoids (Lup (**2**), Derru (**6**), and IsoA (**7**)) from leaves, while 50% ethanol was employed for the extraction of the isoflavonoid glycoside GTG (**4**). Additionally, 68% ethanol was used to extract DerA (**1**), Lup (**2**), Dip (**3**), and Gen (**5**) from stems according to our previous optimization (data not shown). Recovery analysis of HPLC System 1 involved the *D. scandens* leaf extract (0.5 mg/mL, ethanolic extract by maceration with 95% ethanol) spiked with Derru (**6**), IsoA (**7**), and Lup (**2**) standards (25–50 μg/mL). For HPLC System 2 recovery, the *D. scandens* stem extract (1 mg/mL, ethanolic extract by maceration with 50% ethanol) was spiked with GTG (**4**) (10–60 μg/mL). HPLC System 3 recovery was assessed using the *D. scandens* stem extract (0.5 mg/mL, ethanolic extract by maceration with 68% ethanol) spiked with Gen (**5**), DerA (**1**), Dip (**3**), and Lup (**2**) (6.25, 25, and 50 μg/mL). Concentrations of the analytes were determined for the spiked and nonspiked samples, and their recovery was calculated according to the equation:

(1) recovery %=concentration in spiked extract−concentration in nonspiked extracttheoretical spiked concentration×100.

### 2.4. Comparative Extraction of *D. scandens* Leaves and Stems

Leaf and stem samples (150 mg each) were extracted with water and aqueous ethanol (6 mL, 25%–98%, Table 1), facilitated by microwave irradiation (650 W) for 100 s. A broad ethanol range (25%–98%) was explored to achieve wide metabolite coverage based on polarity. Ethanol was selected for its safety and pharmaceutical suitability. The extraction was hypothesis‐driven, targeting polarity‐dependent enrichment: Polar glycosides (e.g., GTG) are better extracted with aqueous ethanol, whereas less polar isoflavones are enriched in higher ethanol concentrations [8]. All extracts were analyzed under identical conditions for systematic comparison. The extract solution was centrifuged at 10, 000 × *g* for 10 min. The residual solid material was subjected to further extraction utilizing the aforementioned procedure three times. The resultant extracts from each sample were pooled, evaporated, and stored at −20°C and analyzed using the respective HPLC systems.

**TABLE 1:** Chemical constituents of *D. scandens* leaves.

Sample ID	Source	Extraction solvents	Extraction yield (% w/w)	Chemical constituents (μg/mg extract)	
				IsoA (7)	Lup (2)
Lf1‐W	Tha Sala, NST	Water	7.18 ± 0.33	ND	ND
Lf1‐25E	Tha Sala, NST	25% ethanol	7.48 ± 0.29	ND	ND
Lf1‐50E	Tha Sala, NST	50% ethanol	7.71 ± 0.22	ND	ND
Lf1‐75E	Tha Sala, NST	75% ethanol	8.25 ± 0.38	3.45 ± 1.07	0.37 ± 0.16
Lf1‐98E	Tha Sala, NST	98% ethanol	8.90 ± 3.09	7.78 ± 1.08	0.98 ± 0.19
Lf2‐98E	Ban Rai, UBP	98% ethanol	14.2 ± 3.0	26.6 ± 3.9	0.61 ± 0.13
Lf3‐98E	Wang Saphung, LOE	98% ethanol	9.52 ± 6.02	10.7 ± 1.1	ND

*Note:* NST, Nakhon Si Thammarat Province; UBP, Ubon Ratchathani Province; LOE, Loei Province.

### 2.5. Comparison of the Anti‐Inflammatory Activity of the Leaf and Stem Extracts on Lipopolysaccharide (LPS)‐Stimulated RAW 264.7 Macrophage Cells

The comparative anti‐inflammatory effects of the *D. scandens* leaf and stem extracts and their chemical constituents, including Derru (**6**), IsoA (**7**), Lup (**2**), GTG (**4**), Gen (**5**), DerA (**1**), and Dip (**3**), were evaluated using RAW 264.7 mouse macrophage cells, as described previously [8]. RAW 264.7 cells were seeded at 5 × 10^4^ cells/well in a 96‐well plate, cultured in DMEM medium containing final concentrations of 10% fetal bovine serum (FBS), 100 U/mL penicillin, and 100 μg/mL streptomycin. The cells were cultured at 37°C and 5% CO_2_ until reaching a confluency of approximately 70%–80%. Untreated cells receiving vehicle (0.1% DMSO in DMEM) served as the baseline control, and cells treated with LPS (0.1 μg/mL) and vehicle served as the negative control. Subsequently, the cells were cotreated with the *D. scandens* extract (12.5–100 μg/mL) and LPS (0.1 μg/mL) for 18 h. For Derru (**6**), IsoA (**7**), and Lup (**2**), investigations were conducted at concentrations ranging from 12.5 to 100 μg/mL, whereas GTG (**4**), Gen (**5**), DerA (**1**), and Dip (**3**) were examined at concentrations ranging from 6.25 to 50 μg/mL. To prepare the vehicle control, DMSO was added to the culture medium to achieve a final concentration of 0.1%, consistent with the preparation of the test substance. The cell culture medium was then removed and replaced with fresh medium containing 0.5 mg/mL 3‐(4,5‐dimethylthiazol‐2‐yl)‐2,5‐diphenyltetrazolium bromide (MTT), and the cells were incubated for 2 h. Following incubation, the medium was removed, and DMSO was added to dissolve the formazan dye, and the absorbance was measured at 595 nm.

*D. scandens* stem and leaf extracts (12.5–100 μg/mL) were evaluated. The leaf metabolites Derru (**6**), IsoA (**7**), and Lup (**2**) were tested at 12.5–50 μg/mL. In contrast, the stem metabolites GTG (**4**) and Gen (**5**) were assessed at 6.25–50 μg/mL, while DerA (**1**) and Dip (**3**) were evaluated at 6.25–25 μg/mL, which maintained cell viability above 80%, and were selected for anti‐inflammatory assays. Cells were cultured to 70%–80% confluence and subsequently treated with the extracts and LPS solution. N^ω^‐nitro‐L‐arginine methyl ester (L‐NAME) 400 μM served as the positive control. Following a 24‐h incubation period, supernatants were collected, and nitric oxide (NO) production was quantified using the Griess reagent, with the absorbance measured at 540 nm. Various concentrations of sodium nitrite (NaNO_2_) were employed to construct a standard calibration curve for quantifying the NO generated in the culture medium. The percentage inhibition of NO production was determined using the following equation:

(2) inhibition of NO %=NOLPS‐treated cell−NOLPS/sample‐treated cell NOLPS−treated cell  ×100.

### 2.6. The Molecular Docking

The present study aimed to evaluate the molecular interactions between isolated bioactive compounds and key inflammation‐associated target proteins. The compounds assessed included IsoA (**7**), Lup (**2**), and Derru (**6**). Their binding affinities were investigated against cyclooxygenase‐1 (COX‐1), cyclooxygenase‐2 (COX‐2), and 5‐lipoxygenase (5‐LOX), which are proposed as therapeutic targets for inflammatory diseases [11].

The X‐ray crystallographic structures of COX‐1 (PDB ID: 3KK6; resolution: 2.75 Å) [12], COX‐2 (PDB ID: 1CX2; resolution: 3.00 Å) [13], and 5‐LOX (PDB ID: 6N2W; resolution: 2.71 Å) [14] were retrieved from the RCSB Protein Data Bank (https://www.rcsb.org/). These protein structures were subsequently prepared using the PDB2PQR web server to assign AMBER force field parameters [15–17]. The protonation states of ionizable residues were determined using PROPKA3 at a simulated physiological pH of 7.4 [18]. Further refinement was conducted with the MolProbity web server to assess and correct steric clashes, thereby improving structural accuracy [19, 20]. The ligands used in this study, including IsoA (**7**) (PubChem CID: 21591148), Lup (**2**) (PubChem CID: 10001388), Derru (**6**) (PubChem CID: 5810067), celecoxib (PubChem CID: 2662), 1‐phenylsulfonamide‐3‐trifluoromethyl‐5‐parabromophenylpyrazole (S58) (PubChem CID: 1396), nordihydroguaiaretic acid (PubChem CID: 688035), ibuprofen (PubChem CID: 3672), and zileuton (PubChem CID: 60490), were retrieved from the PubChem database in SDF format (https://pubchem.ncbi.nlm.nih.gov/). All ligands were subjected to energy minimization using the Merck Molecular Force Field (MMFF94) [21] and subsequently converted to PDBQT format using Open Babel v.2.4.1 [22] in preparation for docking simulations.

Protein and ligand structures were prepared using AutoDock Tools Version 1.5.6. Kollman charges were assigned to all protein structures, while Gasteiger charges were added to all ligands [23]. The grid boxes for COX‐1, COX‐2, and 5‐LOX were generated based on the coordinates reported in previous studies (Table S3) to cover the amino acid residues within the active site of each protein. Docking simulations were carried out using AutoDock 4.2, with the number of genetic algorithm (GA) runs set to 100, while all other parameters were maintained at their default settings [23]. Prior to performing each docking simulation, the docking parameters were validated for each respective protein by redocking the cocrystallized ligands using the defined settings. The accuracy of the redocking was assessed by comparing the redocked ligand conformations with their corresponding cocrystallized structures and calculating the root‐mean‐square deviation (RMSD) values. All ligands achieved RMSD values of less than 2 Å (Figure S3), which is considered acceptable for docking simulations and indicative of reliable binding pose prediction [24, 25]. Subsequently, these validated parameters were applied to the docking of the ligands investigated in this study. The docking results were analyzed using the protein–ligand interaction profiler (PLIP) to evaluate the interactions between the ligands and the amino acid residues of the proteins, with all settings kept at their default values [26, 27]. Figures illustrating the protein–ligand interactions were rendered using PyMOL Version 2.5.2 (Schrödinger, LLC, New York, USA).

### 2.7. Statistical Analysis

Statistical analyses were performed using GraphPad Prism Version 9.1.1 (GraphPad Software, Boston, MA, USA). Statistical differences between treatment groups were evaluated using one‐way analysis of variance (ANOVA) followed by Tukey’s multiple comparison test. A *p* value of less than 0.05 was considered statistically significant. IC_50_ values were calculated separately using nonlinear regression analysis with a four‐parameter logistic model (log[inhibitor] vs. response, variable slope) in GraphPad Prism.

## 3. Results and Discussion

### 3.1. Analytical Performance of the HPLC Systems

**FIGURE 2:**
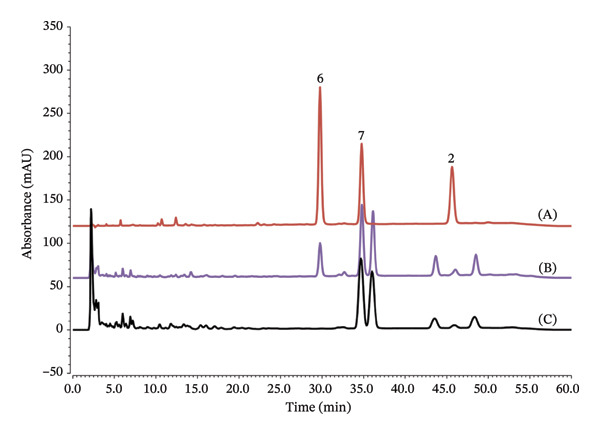
HPLC chromatograms of the isoflavones from *D. scandens* leaves. (A) Reference standards mixture containing derrubone (Derru, **6**), isoangustone A (IsoA, **7**), and lupalbigenin (Lup, **2**) at 200 μg/mL. (B) Analysis of the crude extract of *D. scandens* leaves obtained by maceration with ethanol (1.67 mg/mL). (C) Analysis of the crude extract of *D. scandens* leaves obtained by microwave‐assisted extraction with ethanol.

HPLC–DAD systems were developed for the metabolite analysis of *D. scandens* leaves and stems and subsequently applied for their quantitative analysis. The analytes extracted from the leaves comprised Derru (**6**), Lup (**2**), and IsoA (**7**), which eluted at approximately 30, 35, and 45 mins, respectively (Figure 2). The ethanolic extract of *D. scandens* leaves obtained by maceration contained these analytes. However, there were also three significant peaks adjacent to IsoA (**7**) and Lup (**2**) that remain uncharacterized. Identification of these components, which might affect the biological activity of *D. scandens* leaves, is underway, as it would be necessary for the consistent quality control of a possible standardized preparation. The LODs and LOQs for Derru (**6**), Lup (**2**), and IsoA (**7**) in HPLC System 1 ranged from 0.08 to 0.23 μg/mL and 0.24–0.71 μg/mL, respectively (Table S4). The calibration curve for Derru (**6**) and Lup (**2**) was 0.78–200 μg/mL with *R* ^2^ = 0.9995 and 0.9993, and for IsoA (**7**), it was 0.39–200 μg/mL with *R* ^2^ = 0.9994. Accuracy was confirmed from the recovery rates: Derru (**6**) had 96.0% and 99.2% recovery at 25 and 50 μg/mL, respectively. IsoA (**7**) had 101% and 102% recovery, respectively, and Lup (**2**) had 96.2% and 99.4% recovery, respectively, from the same applied concentrations (Table S5). All recoveries were within the acceptable 95%–105% range, indicating high accuracy. Precision, represented by CV%, was ≤ 0.58%. The low CV values demonstrate excellent precision and repeatability.

For the *D. scandens* stems, GTG (**4**), Gen (**5**), DerA (**1**), Lup (**2**), and Dip (**3**) are the principal anti‐inflammatory metabolites identified [8]. As a disaccharide derivative, GTG (**4**) is relatively more polar than the other constituents. HPLC System 2 was developed and validated for individual GTG (**4**) determination (Figure S4), with LOD and LOQ values of 0.07 and 0.22 μg/mL, respectively (Table S4). The HPLC System 3 analyzed Gen (**5**), DerA (**1**), Dip (**3**), and Lup (**2**) (Figure S5) with LOQ values ranging from 0.41 to 1.05 μg/mL and calibration ranges of 0.78–200 μg/mL for DerA (**1**) and Lup (**2**), and 1.56–200 μg/mL for Gen (**5**) and Dip (**3**) (Table S4). HPLC System 3 exhibits lower sensitivity compared to the published study [8], with LOQs ranging from 0.41 to 1.05 μg/mL versus 0.03–0.18 μg/mL in the published study. However, HPLC System 3 demonstrates broader calibration ranges: 0.78–200 μg/mL for DerA (**1**) and Lup (**2**), and 1.56–200 μg/mL for Gen (**5**) and Dip (**3**), compared to 0.31–40.00 μg/mL in the published study [8]. HPLC System 3 exhibits high analytical precision, with correlation coefficients (*R* ^2^) exceeding 0.999 for all the metabolites, particularly 0.9999 for DerA (**1**), indicating high sensitivity and reliability in detecting and quantifying these compounds at low concentrations. In HPLC System 2, GTG (**4**) showed consistent recovery from 97.8% to 103% across concentrations of 10–60 μg/mL (Table S6). HPLC System 3 exhibited similar recovery for multiple analytes: Gen (**5**) had recovery rates between 95.7% and 104% across 6.25–50 μg/mL concentrations, DerA (**1**) from 95.9% to 104%, Dip (**3**) around 102% across all concentrations, and Lup (**2**) between 93.6% and 103% (Table S7). These recovery values within the 93.6%–104% range indicate excellent accuracy and reliability of the HPLC systems for quantitative analysis. The validated HPLC–DAD systems demonstrated high sensitivity, accuracy, and reproducibility, ensuring reliable quantification of key isoflavones in both leaves and stems.

### 3.2. HPLC Determination of the Isoflavones in the Leaves and Stem Extracts of *D. scandens* From Different Geolocations

Analyses of *D. scandens* leaves collected from various locations in Thailand were conducted, with samples extracted by MAE using different ethanol concentrations as extraction solvents (Table 1). Samples from Tha Sala, Nakhon Si Thammarat (Lf1), were extracted using water and varying ethanol concentrations (25%, 50%, 75%, and 98%), resulting in extraction levels ranging from 7.18% to 8.90%. The metabolites IsoA (**7**) and Lup (**2**) were not detected (ND) in samples using water and 25% and 50% ethanol. However, higher ethanol concentrations demonstrated improved extractive levels, with 75% ethanol yielding 3.45 ± 1.07 μg/mg IsoA (**7**) and 0.37 ± 0.16 μg/mg Lup (**2**), while 98% ethanol extracted 7.78 ± 1.08 μg/mg IsoA (**7**) and 0.98 ± 0.19 μg/mg Lup (**2**). Comparison of the samples from different geographical locations indicated that the Ban Rai sample (Lf2) extracted using 98% ethanol exhibited the highest extraction yield (14.2%) and a significantly higher IsoA (**7**) content (26.6 ± 3.9 μg/mg) with 0.61 ± 0.13 μg/mg Lup (**2**). The Wang Saphung sample (Lf3) extracted with 98% ethanol yielded 9.52% of extract with 10.7 ± 1.1 μg/mg IsoA (**7**). For the *D. scanden*s leaf extract obtained through maceration with 95% ethanol, the metabolites included Derru (**6**), IsoA (**7**), and Lup (**2**) at 30.3 ± 0.1, 113 ± 1, and 11.8 ± 0.1 μg/mg in the extract, respectively. The MAE process resulted in undetectable levels of Derru (**6**) (Figure 2), which may be attributed to the instability of Derru (**6**) under microwave irradiation and heating conditions.

Chemical analysis of *D. scandens* stems obtained from different locations in Thailand, using varying ethanol concentrations as extraction solvents, was performed (Table 2). Samples from Tha Sala, Nakhon Si Thammarat, revealed that higher ethanol concentrations generally yielded higher metabolite levels of GTG (**4**), DerA (**1**), Dip (**3**), and Lup (**2**). The most dramatic increase occurred with 75% ethanol (St1‐75E), which extracted significantly higher amounts of DerA (**1**) (9.30 ± 0.02 μg/mg), Dip (**3**) (20.9 ± 0.2 μg/mg), and Lup (**2**) (40.4 ± 0.1 μg/mg) compared to the lower ethanol concentrations. When comparing samples using 98% ethanol across different locations of *D. scandens* stem collections, there were notable variations. The Ban Rai, UBP sample (St2‐98E) showed relatively low GTG (**4**) content (6.88 ± 0.01 μg/mg) but a high Lup (**2**) content (150 ± 2 μg/mg). The Nawah, UBP sample (St4‐98E) demonstrated the highest concentrations overall, particularly for DerA (**1**) (103 ± 1 μg/mg) and Lup (**2**) (296 ± 6 μg/mg). Again, the geographical location significantly influenced the chemical composition of *D. scanden* stems. Environmental factors such as soil composition, rainfall, and temperature have been shown to alter secondary metabolite accumulation. The water‐only extraction (St1‐W) yielded the lowest concentrations of all constituents, indicating that ethanol is effective at extracting these metabolites. This observation has direct implications for future standardization, as both solvent choice and geographical sourcing must be optimized to ensure consistent phytochemical quality for medicinal use.

**TABLE 2:** Chemical constituents of *D. scandens* stems.

Sample ID	Source	Extraction solvents	Chemical constituents (μg/mg extract)			
			GTG (4)	DerA (1)	Dip (3)	Lup (2)
St1‐W	Tha Sala, NST	Water	11.0 ± 1.2	0.10 ± 0.02	0.25 ± 0.06	0.49 ± 0.09
St1‐25E	Tha Sala, NST	25% ethanol	11.8 ± 0.1	0.12 ± 0.01	0.34 ± 0.02	0.48 ± 0.01
St1‐50E	Tha Sala, NST	50% ethanol	17.6 ± 0.1	0.44 ± 0.00	0.40 ± 0.00	0.75 ± 0.00
St1‐75E	Tha Sala, NST	75% ethanol	21.8 ± 0.6	9.30 ± 0.02	20.9 ± 0.2	40.4 ± 0.1
St1‐98E	Tha Sala, NST	98% ethanol	27.0 ± 1.5	8.26 ± 0.11	17.6 ± 0.2	32.9 ± 0.4
St2‐98E	Ban Rai, UBP	98% ethanol	6.88 ± 0.01	7.03 ± 0.18	69.7 ± 1.0	150 ± 2
St3‐98E	Song Yang, UBP	98% ethanol	16.8 ± 0.2	11.4 ± 0.2	31.1 ± 0.5	53.2 ± 2.1
St4‐98E	Nawah, UBP	98% ethanol	25.0 ± 0.2	103 ± 1	(2.00 ± 0.189) × 10^2^	296 ± 6

*Note:* NST, Nakhon Si Thammarat Province; UBP, Ubon Ratchathani Province.

The study reveals significant differences in the Lup (**2**) content between *D. scandens* leaves and stems. Leaf extracts showed lower Lup (**2**) concentrations, ranging from 0.61 to 0.98 μg/mg in 98% ethanol extracts (Table 1). Stem extracts exhibited substantially higher Lup (**2**) concentrations, varying widely depending on the extraction method and the geographic location, with values from 32.9 to 296 μg/mg in the 98% ethanol extracts (Table 2). This comparison shows that *D. scandens* stems contain significantly higher Lup (**2**) concentrations than the leaves, with stem extracts having up to 25 times more Lup (**2**) than the highest leaf extract concentration. To our knowledge, this is the first systematic comparison of isoflavone profiles across geographical locations in *D. scandens* leaves and stems using validated HPLC–DAD, highlighting the impact of both extraction conditions and origin on metabolite accumulation.

### 3.3. Comparative Cytotoxicity and Anti‐Inflammatory Effects of the Isolated Metabolites and Extracts of *D. scandens* Leaves and Stems

Derru (**6**), IsoA (**7**), and Lup (**2**) demonstrated concentration‐dependent mild cytotoxic effects in RAW 264.7 cells. At concentrations ranging from 12.5 to 50 μg/mL, cell viability remained at a minimum of 81% compared to the control (Figure S6A). Increasing the concentration to 100 μg/mL decreased cell viability to less than 80% compared to the untreated control (0.1% DMSO in culture medium). Specifically, Derru (**6**), IsoA (**7**), and Lup (**2**) exhibited cell viability rates of only 5.7%, 9.75%, and 12.7%, respectively. Regarding the metabolites from the stem, DerA (**1**) and Dip (**3**) at 6.25–25 μg/mL resulted in cell viability of more than 80%, whereas Gen (**5**) and GTG (**4**) at 6.25–50 μg/mL maintained cell viability at a minimum of 97%. The concentration of the investigated compounds which afforded at least 80% cell viability at a given dose was evaluated for their inhibitory activity of NO secretion in LPS‐induced RAW 264.7 cells.

**FIGURE 3:**
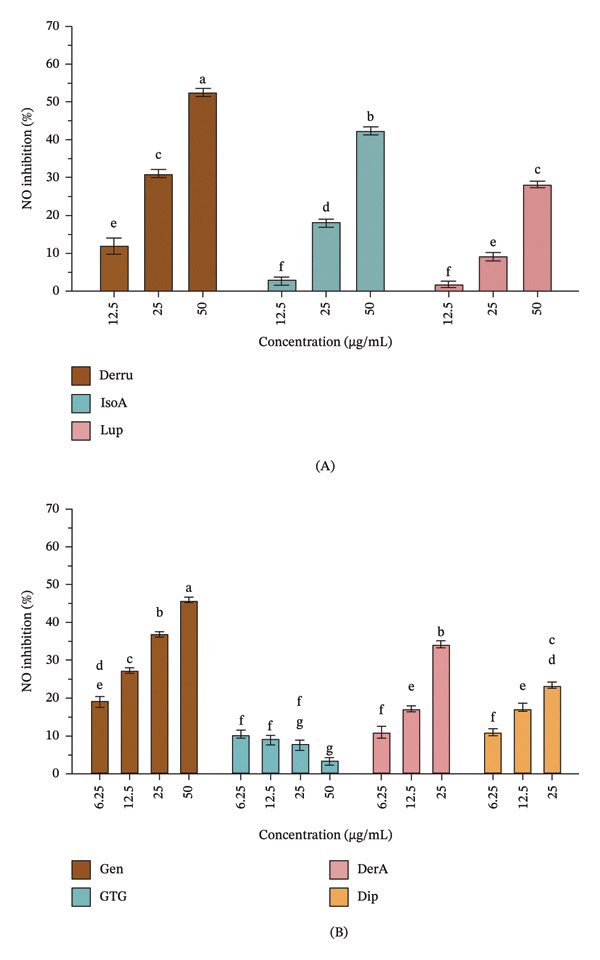
Effects of the metabolites of *D. scandens* leaves and stems on the inhibition of NO secretion in LPS‐induced RAW 264.7 macrophage cells: (A) chemical constituents from the leaves and (B) chemical constituents from the stems (both leaves and stems contain Lup (**2**)). The metabolites are derrubone (Derru, **6**), isoangustone A (IsoA, **7**), lupalbigenin (Lup, **2**), genistein (Gen, **5**), genistein‐7‐*O*‐[2]‐β‐glucopyranoside (GTG, **4**), derrisisoflavone A (DerA, **1**), and 6,8‐diprenylgenistein (Dip, **3**).

Derru (**6**), IsoA (**7**), Lup (**2**), Gen (**5**), and DerA (**1**) inhibited NO secretion in a concentration‐dependent manner (Figure 3A and B). GTG (**4**) produced a low inhibition of NO secretion and a low inhibition of eicosanoid production in vitro [4, 8]; no concentration‐dependent outcome was observed. To compare the NO secretion inhibition at the same concentration (25 μg/mL), the inhibition of NO secretion by DerA (**1**) (34.3%) and Dip (**3**) (23.3%) from the stem was higher compared to Derru (**6**) (31.0%) and IsoA (**7**) (18.0%) from the leaves (Figure 3) (Table S8). Lup (**2**) found in both leaves and stems, at 25 and 50 μg/mL, demonstrated inhibition of NO secretion of 9.17% and 28.1%, respectively (Table S8). A previous investigation of the anti‐inflammatory properties of the metabolites from *D. scandens* stems also demonstrated a dose‐dependent inhibition of NO secretion in LPS‐induced RAW 264.7 cells [8]. At 25 μM, the order of inhibition of NO production was as follows: Gen (5) (55.3%) > Lup (2) (44.6%) > DerA (1) (30.2%) > Dip (3) (24.2%) > GTG (4) (2.5%) [8]. As positive controls, dexamethasone, a synthetic glucocorticoid with potent anti‐inflammatory activity, exhibited a concentration‐dependent inhibition of NO production in LPS‐induced RAW 264.7 macrophage cells. At concentrations of 3.13, 6.25, and 12.5 μM, dexamethasone inhibited NO secretion by 27.37%, 35.58%, and 36.17%, respectively. Meanwhile, L‐NAME, a nonselective nitric oxide synthase (NOS) inhibitor, exhibited a higher NO inhibitory effect, reaching 46.82% inhibition at a concentration of 400 μM. These reference values provide benchmarks for evaluating the anti‐inflammatory potential of *D. scandens* extracts and metabolites. This study demonstrated the inhibitory activity of NO secretion in LPS‐induced RAW 264.7 cells by the metabolites from *D. scandens* leaves, indicating them to be a possibly more sustainable source of anti‐inflammatory compounds.

Derru (**6**) and IsoA (**7**) have been reported to exhibit additional bioactivities beyond their anti‐inflammatory effects. Derru (**6**) has been identified as a potent Hsp90 inhibitor with significant antiproliferative activity in breast cancer models [28, 29]. IsoA (**7**) has demonstrated cytotoxic and tumor‐suppressive effects in colorectal, prostate, and melanoma models, as well as protective effects in diabetic nephropathy through modulation of inflammatory and fibrotic pathways [30–33]. Notably, several of these reported activities involve regulation of inflammatory signaling pathways, supporting the biological relevance of the anti‐inflammatory effects observed in the present study. Therefore, Derru (**6**), IsoA (**7**), and *D. scandens* leaf extracts merit further investigation in inflammation‐related conditions. Further preclinical and clinical studies are required to confirm their safety and efficacy.

The effects of the *D. scandens* leaf and stem extracts on cell viability were investigated in the range 12.5–100 μg/mL. For the Lf1 extract and the leaf samples from other sources (Lf2 and Lf3), cell viability was maintained at a minimum of 84% (Figure S7). Similarly, for the stem (St1) extract at 12.5–100 μg/mL, cell viability of at least 84% was observed. However, for extracts of the stem sources St2 and St4 at 100 μg/mL, cell viability decreased to 21.1% and 14.4%, respectively (Figure S7D).

**FIGURE 4:**
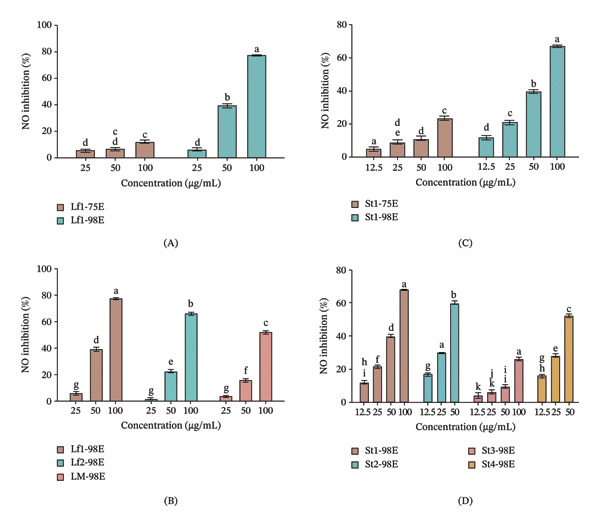
Inhibition of NO production in LPS‐stimulated RAW 264.7 macrophages by *D. scandens* extracts. (A) Leaf extracts (Lf1) obtained using different ethanol concentrations. (B) Leaf extracts from different locations obtained using 98% ethanol. (C) Stem extracts (St1) obtained using different ethanol concentrations. (D) Stem extracts from different locations obtained using 98% ethanol. Data are presented as mean ± SEM (*n* = 3).

At the concentrations of the leaf and stem extracts that retain over 80% cell viability, the extracts were evaluated for the inhibition of NO secretion in LPS‐induced RAW 264.7 macrophage cells. For both the stem and leaf extracts, the inhibition of NO secretion increased when higher ethanol concentrations were used for extraction. Extracts of both the stems and leaves obtained using water to 50% ethanol did not inhibit NO secretion. The findings are consistent with the chemical analyses presented in Tables 1 and 2, which indicate that lower ethanol concentrations were inadequate for extracting significant quantities of the bioactive isoflavones, including IsoA (**7**), Lup (**2**), DerA (**1**), and Dip (**3**). These metabolites, recognized for their inhibition of NO secretion, were detected in higher concentrations only when 75%–98% ethanol was employed as the extraction solvent. The leaf and stem extracts obtained using 98% ethanol demonstrated the highest inhibition of NO secretion (Figure 4A and C). In the case of the leaf extracts, the inhibition of NO secretion correlated with the overall yields of the constituents (Table 1), where 98% ethanol produced the highest yields of IsoA (**7**) and Lup (**2**). For the stem extracts, the 75% ethanol extract produced the highest yields of DerA (**1**), Dip (**3**), and Lup (**2**), although the 98% ethanol extract exhibited the highest level of NO secretion inhibition. The metabolites responsible for the enhanced NO secretion in the 98% ethanol‐derived extract of the leaves remain to be established before a standardized protocol can be developed.

Comparison of the leaf materials from the different geographic locations indicated that Lf1 exhibited the highest inhibition of NO secretion (77.4% at 100 μg/mL), which was greater than Lf2 (66.0% inhibition) and Lf3 (52.4% inhibition) at the same concentration (Table S9). However, the Lf2 extract contained the highest concentration of IsoA (**7**), indicating that some unidentified constituents must also contribute to the inhibition of NO secretion. In the case of the stems at 50 μg/mL, St2 produced the highest inhibition of NO secretion. When extracted with 98% ethanol and tested at a fixed extract concentration of 50 μg/mL, the leaf and stem extracts produced inhibition of NO in the ranges of 6.59%–39.2% and 9.44%–59.6%, respectively (Table S9). Consequently, the stem segment of *D. scandens* may exhibit a greater inhibition of NO secretion in LPS‐induced RAW 264.7 macrophage cells. However, the chemical composition of the stem and leaf extracts varies depending on geographical location. Additionally, the age of the plant may influence both the metabolite profile quality and yield quantity, which remains to be explored.

This study provides a comparison of the effects of the metabolites and different extraction protocols applied to the *D. scandens* leaves and stems on the inhibition of NO secretion, based on the geographical location of origin and the solvent extraction polarity. The objective was to determine whether utilizing the leaves instead of the stems could potentially be a more sustainable resource [34]. The present study identified the presence of bioactive compounds Derru (**6**), IsoA (**7**), and Lup (**2**) in the leaves of *D. scandens*, which have been shown to possess anti‐inflammatory properties. However, the leaf and stem materials have different chemical profiles. Thus, the leaves cannot be directly substituted for the stems, which have been traditionally utilized, are approved, and have an established safety profile in patients, since the stems have DerA (**1**), Lup (**2**), Dip (**3**), and Gen (**5**) as the bioactive components [8, 35]. Further research is necessary for the development of standardized extraction methods and quality control measures to ensure consistent bioactive compound content in the leaf extracts, and more comprehensive studies on the long‐term sustainability, safety, efficacy, and consistency of leaf extracts in comparison to the stem extracts are needed. Studies of the mechanism of the anti‐inflammatory effects of the most active isoflavones are also warranted. Long‐term studies evaluating the pharmacodynamics and safety profiles of leaf extracts in preclinical models will be crucial to advancing their therapeutic potential.

This study aimed to compare the metabolites and anti‐inflammatory activity of the leaves and stems of *D. scandens* in consideration for the development of a more sustainable source than the approved stem preparation. Derru (**6**), Iso (**7**), and Lup (**2**) were isolated from *D. scandens* leaves. HPLC–DAD systems were developed and validated for the quantitative analysis of the isoflavones in the leaf and stem extracts. A higher ethanol concentration increased the extraction yields of the target compounds Derru (**6**), IsoA (**7**), Lup (**2**), DerA (**1**), and Dip (**3**). The geographical origin of the respective plant materials significantly influenced the metabolite profiles of the leaves and stems. Stem extracts contained higher levels of the target compounds, which correlated with a stronger inhibition of NO secretion in LPS‐induced RAW 264.7 macrophage cells compared to the leaf extracts. These findings suggest that while both *D. scandens* leaves and stems inhibit NO secretion in LPS‐induced RAW 264.7 macrophage cells, the stem portion demonstrates higher potency and cannot be directly substituted by a leaf preparation without further research to examine toxicity since the bioactive metabolites are different.

### 3.4. The Molecular Docking

The molecular docking results are summarized in Table S10. For COX‐1, the cocrystallized ligand, celecoxib, showed the highest binding energy of −12.06 kcal/mol, forming hydrogen bonds with LEU352, SER516, ILE517, and PHE518 (Figure S8A). Among the test compounds, Lup (**2**) exhibited the highest binding energy of −8.76 kcal/mol, forming a hydrogen bond with MET522 and hydrophobic interactions with VAL116, LEU352, TYR355 (two interactions), LEU359, PHE381, TYR385, TRP387, PHE518, ALA527, and LEU531 (Figure S8C). Derru (**6**) demonstrated a binding energy of −8.11 kcal/mol, forming hydrogen bonds with ARG120 (two interactions), MET522, and ALA527 (Figure S8D). IsoA (**7**) displayed a binding energy of −8.06 kcal/mol, forming hydrogen bonds with MET522 (two interactions) (Figure S8B). Notably, all three tested compounds formed hydrogen bonds with the common residue MET522. The reference compound, ibuprofen (nonselective COX inhibitor), exhibited a binding energy of −7.80 kcal/mol. It formed hydrogen bonds with GLN192, ILE517, and PHE518, in addition to several hydrophobic interactions (Figure S8E). In the present study, ibuprofen demonstrated a higher binding affinity for COX‐1 compared to COX‐2, which is consistent with previous in vitro findings reporting its preferential inhibition of COX‐1 [36, 37]. The hydrogen bonding and hydrophobic interactions observed for the tested compounds involved key residues within the COX‐1 active site, including HIS90, ARG120, GLN192, TYR355, TYR385, ILE434, HIS513, SER516, PHE518, ILE523, and SER530 [38].

For COX‐2, the cocrystallized ligand S58 (celecoxib analog) exhibited the highest binding energy of −11.24 kcal/mol, forming hydrogen bonds with GLN192, ILE517, and PHE518 (Figure S9A). Among the test compounds, Lup (**2**) showed the highest binding energy of −8.98 kcal/mol, forming hydrogen bonds with GLN192, SER353, ILE517, and PHE518, along with hydrophobic interactions with VAL349 (two interactions), ALA516, ILE517, PHE518, VAL523, ALA527, and LEU531 (Figure S9C). Derru (**6**) and IsoA (**7**) showed binding energies of −6.89 and −5.61 kcal/mol, respectively. Derru (**6**) formed hydrogen bonds with ARG120, TYR355, and ALA527 (Figure S9D), while IsoA (**7**) formed hydrogen bonds with LYS83, ARG120, LEU352, SER353, and GLU524 (Figure S9B). Interestingly, the absence of a hydroxyl group at the C‐3′ position of Ring B in Lup (**2**) appears to contribute to its enhanced binding affinity compared to IsoA (**7**), which retains a hydroxyl group at that position. Structure–activity relationship (SAR) analysis indicates that substitution with hydroxyl and prenyl groups at specific positions on Rings A and B of the flavonoid scaffold significantly improves anti‐inflammatory activity [39]. The reference compound ibuprofen exhibited a binding energy of −6.36 kcal/mol, forming hydrogen bonds with TYR355 and a salt bridge interaction with ARG120 (Figure S9E), consistent with previously reported findings [40]. The key residues involved in hydrogen bonding and hydrophobic interactions with the tested compounds were located within the COX‐2 active site and included HIS90, ARG120, GLN192, TYR355, TYR385, VAL434, ARG513, ALA516, PHE518, VAL523, and SER530 [38]. Previous studies reported that chrysin, a flavonoid, inhibited COX‐2 with an IC_50_ of 18.48 μM, and docking analysis showed hydrogen bonds with ARG120, VAL349, and SER353, along with several hydrophobic interactions [41]. NPC7, a synthetic flavonoid, showed 99.24% inhibition of COX‐2, consistent with hydrogen bonding to SER530 and hydrophobic interactions within the active site [42]. Additionally, flavonoid glycoside 7‐*O*‐methyl aromadendrin formed hydrogen bonds with TYR355, LEU453, and SER353 in docking studies, consistent with its COX‐2 inhibition activity (IC_50_ = 2.55 ± 0.02 μg/mL) [43].

Regarding 5‐LOX, the cocrystallized ligand 30Z (nordihydroguaiaretic acid) exhibited a binding energy of −5.10 kcal/mol, forming hydrogen bonds with PRO569, ARG596, and ILE673 (two interactions) (Figure S10A). Among the test compounds, Lup (**2**) showed the highest binding energy of −7.55 kcal/mol, forming hydrogen bonds with GLN363, ARG596 (two interactions), and ILE673, along with hydrophobic interactions with PHE359 (two interactions), GLN363, LEU368 (two interactions), ALA410, TRP599, ALA603, and LEU607 (Figure S10C). IsoA (**7**) and Derru (**6**) followed, with binding energies of −7.45 and −6.32 kcal/mol, respectively. IsoA (**7**) formed hydrogen bonds with GLN363, PRO569, and ARG596 (two interactions) (Figure S10B), while Derru (**6**) formed hydrogen bonds with GLN363, LEU368, ARG596 (two interactions), and ILE673 (Figure S10D). The positive control, zileuton, showed a binding energy of −5.45 kcal/mol, forming hydrogen bonds with GLN363 (two interactions) and hydrophobic interactions with LEU368, ASN407, ALA410, ARG411, and LEU607 (Figure S10E). These findings are consistent with previous studies demonstrating that zileuton forms hydrogen bonds via its amine NH_2_ group with GLN363 [44], as well as hydrophobic interactions with PHE177, LEU368, HIS372, ILE406, ASN407, ALA410, LEU414, PHE421, LEU607, and ILE67 [45]. The hydrogen bonding and hydrophobic interactions observed in the test compounds involved key residues within the 5‐LOX active site, including PHE177, TYR181, HIS367, LEU368, HIS372, ASN407, ALA410, LEU414, PHE421, HIS550, LEU607, and ILE673 [45].

Based on the molecular docking results, Lup (**2**) exhibits promising potential as a dual inhibitor of COX and LOX enzymes, indicating its possible development as a therapeutic agent for inflammatory diseases.

## 4. Conclusions

This study aimed to comparatively analyze the chemical constituents and anti‐inflammatory activity of *D. scandens* leaves and stems. Derru (**6**), IsoA (**7**), and Lup (**2**) were isolated from *D. scandens* leaves. HPLC–DAD systems were developed and validated for the quantitative analysis of the isoflavones in the leaf and stem extracts. The extraction solvent concentration influences the compounds that are extracted; the more increased ethanol concentration, the more increased extraction of target compounds: Derru (**6**), IsoA (**7**), Lup (**2**), DerA (**1**), and Dip (**3**). Geographical location significantly influenced the chemical composition of both the leaves and stems. Stem extracts contain a higher amount of target compounds and generally exhibit higher inhibition of NO secretion in LPS‐induced RAW 264.7 macrophage cells compared to the leaf extracts. The findings suggest that while both *D. scandens* leaves and stems contain compounds inhibiting NO secretion in LPS‐induced RAW 264.7 macrophage cells, the stem portion demonstrates higher potency and cannot be substituted by the leaves. Further studies on standardization, safety, and efficacy of leaf extracts are needed to support their therapeutic use.

## Author Contributions

Benyatip Buajan: conceptualization, methodology, investigation, visualization, formal analysis, resources, and writing–original draft; Mudtorlep Nisoa: methodology, resources, and writing–review and editing; Fonthip Makkliang: conceptualization, methodology, investigation, and writing–review and editing; Atthaphon Konyanee: conceptualization, methodology, investigation, and writing–review and editing; Waraporn Putalun: conceptualization, methodology, and writing–review and editing; Geoffrey A. Cordell: writing–review and editing; Rawiwan Charoensup: writing–review and editing; and Gorawit Yusakul: conceptualization, methodology, investigation, visualization, formal analysis, resources, writing–original draft, writing–review and editing, supervision, project administration, and funding acquisition.

## Funding

This research was supported by the Research Fund for Graduate Students (Fiscal Year 2023; CGS‐RF‐2023/09) and the High‐Potential Candidates Scholarship Program (Contract No. HP013/2021), Walailak University, Nakhon Si Thammarat, Thailand. The authors express their gratitude for the facilities and support provided by The Center for Scientific and Technological Equipment, Walailak University, Nakhon Si Thammarat, Thailand. Furthermore, the authors acknowledge the support from the Hub of Knowledge in Microwave Heating and Applications, Walailak University, Nakhon Si Thammarat 80160, Thailand.

## Disclosure

All authors approved the version to be published.

## Ethics Statement

This article does not contain any studies with animals or human participants performed by any of the authors. The protocols below relate to the cell culture and treatment methods used in the research project “Leaves of *Derris scandens* (Roxb.) Benth. extraction technology for the improvement of anti‐inflammation activity and development as HDES‐based microemulsions formulation.” This study was approved by the Institutional Biosafety Committee of Walailak University. Approval under clearance no. WU‐IBC‐66‐050 was effective from November 30, 2023, to May 31, 2024.

## Conflicts of Interest

The authors declare no conflicts of interest.

## Supporting Information

Additional supporting information can be found online in the Supporting Information section.

## Supporting information


**Supporting Information** Figure S1. ^1^H‐NMR spectrum of derrubone. Figure S2. ^1^H‐NMR spectrum of isoangustone A. Figure S3. (A) Root‐mean‐square deviation (RMSD) values between redocked and cocrystallized ligands for celecoxib (CEL). (B) 1‐Phenylsulfonamide‐3‐trifluoromethyl‐5‐parabromophenylpyrazole (S58) and (C) nordihydroguaiaretic acid (30Z) within their respective protein crystal structures. Yellow structures represent redocked ligands, while purple structures represent the cocrystallized ligands. Figure S4. HPLC chromatograms of (1) genistein‐7‐*O*‐[2]‐β‐glucopyranoside (GTG, **4**) at 260 nm; A and B are reference standards of GTG (**4**) (200 μg/mL) and the extract of *D. scandens* leaves (3.33 mg/mL), respectively. Figure S5. HPLC chromatograms of reference compounds including (1) genistein (Gen, **5**), (2) derrisisoflavone A (DerA, **1**), (3) lupalbigenin (Lup, **2**), and (4) 6,8‐diprenylgenistein (Dip, **3**); A and B are reference isoflavone derivatives (200 μg/mL) and *D. scandens* stem extracts obtained with 68% ethanol (1.0 mg/mL), respectively. Figure S6. Effect of the metabolites of *D. scandens* leaves and stems on cell viability in LPS‐induced RAW 264.7 macrophage cells: (A) metabolites from the leaves and (B) metabolites from the stems (both the leaves and stems contain lupalbigenin (Lup, **2**). Derrubone (Derru, **6**), isoangustone A (IsoA, **7**), lupalbigenin (Lup, **2**), genistein (Gen, **5**), genistein‐7‐*O*‐[α‐rhamnopyranosyl‐(1 ⟶ 6)]‐β‐glucopyranoside (GTG, **4**), derrisisoflavone A (DerA, **1**), and 6,8‐diprenylgenistein (Dip, **3**) were characterized from the leaves and stems as indicated in the text. Figure S7. Effects of *D. scandens* leaf and stem extracts on cell viability in lipopolysaccharide‐induced RAW 264.7 macrophage cells. (A) Leaf material extracted with water and 25%–98% ethanol, (B) leaf extracts from different geographic sources (Lf1–Lf3), and (C) stems extracted with water and 25%–98% ethanol, and (D) extracts of the stems from different geographic sources (St1–St4). Figure S8. Interactions between ligands and amino acid residues in the active site of COX‐1 (PDB ID: 3KK6). (A) The ligands include CEL, the cocrystallized ligand, (B) isoangustone A (IsoA, **7**), (C) lupalbigenin (Lup, **2**), (D) derrubone (Derru, 6), and (E) ibuprofen. Ligands are shown in ball‐and‐stick models, with heteroatoms colored as follows: carbon (C) in orange, oxygen (O) in red, nitrogen (N) in blue, sulfur (S) in yellow, fluorine (F) in cyan, and hydrogen (H) in white. Amino acid residues are depicted as line models with heteroatoms labeled according to the same color scheme. The protein backbone is shown as a wheat‐colored ribbon. Yellow dashed lines indicate hydrogen bonds, grey dashed lines indicate hydrophobic interactions, and green dashed lines represent *π*−*π* stacking interactions between ligand atoms and amino acid residues. Figure S9. Interactions between ligands and amino acid residues in the active site of COX‐2 (PDB ID: 1CX2). (A) The ligands include S58, the cocrystallized ligand, (B) isoangustone A (IsoA, 7), (C) lupalbigenin (Lup, 2), (D) derrubone (Derru, 6), and (E) ibuprofen. Ligands are shown in ball‐and‐stick models, with heteroatoms colored as follows: carbon (C) in orange, oxygen (O) in red, nitrogen (N) in blue, sulfur (S) in yellow, fluorine (F) in cyan, bromine (Br) in ruby red, and hydrogen (H) in white. Amino acid residues are depicted as line models with heteroatoms labeled according to the same color scheme. The protein backbone is shown as a wheat‐colored ribbon. Yellow dashed lines indicate hydrogen bonds, grey dashed lines indicate hydrophobic interactions, and blue dashed lines indicate salt bridge interactions between ligand atoms and amino acid residues. Figure S10. Interactions between ligands and amino acid residues in the active site of 5‐LOX (PDB ID: 6 N2W). (A) The ligands include 30Z, the cocrystallized ligand, (B) isoangustone A (IsoA, **7**), (C) lupalbigenin (Lup, **2**), (D) derrubone (Derru, **6**), and (E) zileuton. Ligands are shown in ball‐and‐stick models, with heteroatoms colored as follows: carbon (C) in orange, oxygen (O) in red, nitrogen (N) in blue, sulfur (S) in yellow, and hydrogen (H) in white. Amino acid residues are depicted as line models with heteroatoms labeled according to the same color scheme. The protein backbone is shown as a wheat‐colored ribbon. Yellow dashed lines indicate hydrogen bonds, grey dashed lines indicate hydrophobic interactions, and green dashed lines represent *π*−*π* stacking interactions between ligand atoms and amino acid residues. The catalytic iron (Fe) is shown as a ruby‐red sphere.

## Data Availability

The data that support the findings of this study are available in the supporting information of this article. Further data are available from the corresponding author upon reasonable request.
