# Supplementary material for: Phytochemical and Geographical Comparison of Derris scandens (Roxb.) Benth. Leaves and Stems and Their Nitric Oxide Production Inhibitory Activity
Source: Scientifica (Cairo). 2026 May 29;2026:2028051. doi: 10.1155/sci5/2028051 (PMC13239046; doi:10.1155/sci5/2028051)
Supplement: Supplementary file 1 — Supporting Information Figure S1. 1H‐NMR spectrum of derrubone. Figure S2. 1H‐NMR spectrum of isoangustone A. Figure S3. (A) Root‐mean‐square deviation (RMSD) values between redocked and cocrystallized ligands for celecoxib (CEL). (B) 1‐Phenylsulfonamide‐3‐trifluoromethyl‐5‐parabromophenylpyrazole (S58) and (C) nordihydroguaiaretic acid (30Z) within their respective protein crystal structures. Yellow structures represent redocked ligands, while purple structures represent the cocrystallized ligands. Figure S4. HPLC chromatograms of (1) genistein‐7‐O‐[2]‐β‐glucopyranoside (GTG, 4) at 260 nm; A and B are reference standards of GTG (4) (200 μg/mL) and the extract of D. scandens leaves (3.33 mg/mL), respectively. Figure S5. HPLC chromatograms of reference compounds including (1) genistein (Gen, 5), (2) derrisisoflavone A (DerA, 1), (3) lupalbigenin (Lup, 2), and (4) 6,8‐diprenylgenistein (Dip, 3); A and B are reference isoflavone derivatives (200 μg/mL) and D. scandens stem extracts obtained with 68% ethanol (1.0 mg/mL), respectively. Figure S6. Effect of the metabolites of D. scandens leaves and stems on cell viability in LPS‐induced RAW 264.7 macrophage cells: (A) metabolites from the leaves and (B) metabolites from the stems (both the leaves and stems contain lupalbigenin (Lup, 2). Derrubone (Derru, 6), isoangustone A (IsoA, 7), lupalbigenin (Lup, 2), genistein (Gen, 5), genistein‐7‐O‐[α‐rhamnopyranosyl‐(1 ⟶ 6)]‐β‐glucopyranoside (GTG, 4), derrisisoflavone A (DerA, 1), and 6,8‐diprenylgenistein (Dip, 3) were characterized from the leaves and stems as indicated in the text. Figure S7. Effects of D. scandens leaf and stem extracts on cell viability in lipopolysaccharide‐induced RAW 264.7 macrophage cells. (A) Leaf material extracted with water and 25%–98% ethanol, (B) leaf extracts from different geographic sources (Lf1–Lf3), and (C) stems extracted with water and 25%–98% ethanol, and (D) extracts of the stems from different geographic sources (St1–St4). Figure S8. Int [file SCI5-2026-2028051-s001.docx]

**Supplementary Materials**

**Phytochemical and Geographical Comparison of *Derris scandens* (Roxb.) Benth. Leaves and Stems and Their Nitric Oxide Production Inhibitory Activity**

## Author information

Benyatip Buajan^1,2^, Mudtorlep Nisoa^3,4,5^, Fonthip Makkliang^4,5,6^_,_ Atthaphon Konyanee^7,8^, Waraporn Putalun^9^_,_ Geoffrey A. Cordell^10, 11^, Rawiwan Charoensup^12,13^, Gorawit Yusakul^1,5,14,15*^

^1^School of Pharmacy, Walailak University, Nakhon Si Thammarat 80160, Thailand

^2^School of Public Health, Walailak University, Nakhon Si Thammarat 80160, Thailand

^3^Futuristic Science Research Center, School of Science, Walailak University, Nakhon Si Thammarat 80160, Thailand

^4^Functional Materials and Nanotechnology Center of Excellence, Walailak University, Nakhon Si Thammarat 80160, Thailand

^5^Hub of Knowledge in Microwave Heating and Applications, Walailak University, Nakhon Si Thammarat 80160, Thailand

^6^School of Languages and General Education, Walailak University, Nakhon Si Thammarat 80160, Thailand

^7^School of Medicine, Walailak University, Nakhon Si Thammarat 80160, Thailand

^8^Research Center in Tropical Pathobiology, Walailak University, Nakhon Si Thammarat 80160, Thailand

^9^Faculty of Pharmaceutical Sciences, Khon Kaen University, Khon Kaen 40002, Thailand

^10^Natural Products Inc., Evanston, IL,60201, USA

^11^Department of Pharmaceutics, College of Pharmacy, University of Florida, Gainesville, FL 32610, USA

^12^Medicinal Plants Innovation Center, Mae Fah Luang University, Chiang Rai 57100, Thailand

^13^School of Integrative Medicine, Mae Fah Luang University, Chiang Rai 57100, Thailand

^14^Department of Pharmaceutical Chemistry and Pharmacognosy, Faculty of Pharmaceutical Sciences, Naresuan University, Phitsanulok 65000, Thailand

^15^Research and Innovation Cluster for Natural Health Products, Naresuan University, Phitsanulok 65000, Thailand

***Corresponding author**

Department of Pharmaceutical Chemistry and Pharmacognosy, Faculty of Pharmaceutical Sciences, Naresuan University, Phitsanulok 65000, Thailand

Email: gorawity@nu.ac.th Tel.: +66 5596 1869

Extraction and isolation of *D. scandens* leaf metabolites

Dried *D. scandens* leaves (Lf2, 5 kg) were macerated twice with 95% ethanol (15 L) for five days at ambient temperature and then filtered. The leaf extract was concentrated at 40°C to afford an ethanolic extract (77.29 g) which was dissolved in hexane and ethyl acetate (1:3, 300 mL) and partitioned four times with water (200 mL each). The solvent phases were evaporated, and the partitioned extract (34.61 g) was fractionated by silica gel column chromatography (95 g Si gel), eluting with hexane and hexane:ethyl acetate mixtures (6:1, 4:1, 3:1, 2:1, and 1:1) at 15 mL/min, yielding 36 fractions (C1F1–F36). The metabolites present in each fraction were analyzed by TLC eluting with hexane:ethyl acetate (1:1) and high-performance liquid chromatography using HPLC system-1 as described in “HPLC-DAD analytical methods for *D. scandens* extracts”).

Fractions C1F1–F16 were further separated using a COSMOSIL C18-OPN (Nacalai Tesque, Inc., Nakagyo-ku, Kyoto, Japan) column eluted with 60% acetonitrile, to yield 75 fractions (C2F1–F75). C2F1–F38 were combined and further subjected to silica gel column chromatography. A mixture of hexane:ethyl acetate (9:1) was utilized as the mobile phase, resulting in 27 fractions (C3F1–F27). The fractions C3F1–F4 and C3F5–7 afforded crystals of Lup (**2**) (96.2 mg) and compound A (66.5 mg), respectively.

Fractions C2F39–74 were combined and subjected to silica gel column chromatography eluted with hexane:ethyl acetate (5:1 to 4:1) to yield 6 fractions (C4F1–F6). Fractions C4F1–F4 were combined and subjected to silica gel column chromatography, eluted with hexane:ethyl acetate (9:1), and compound B (47.4 mg) was obtained after solvent evaporation of fractions F74–F98.

Structure elucidation of the isolated compounds from *D. scandens* leaves

Compound A (derrubone (Derru, **6**), C_21_H_18_O_6_, colorless, amorphous solid) was isolated. The ^1^H NMR spectrum showed aromatic proton signals [δ 7.91 (s), δ 6.31 (brs), δ 7.05 (d, 1.5), δ 6.88 (d, 8.0), and δ 6.95 (dd, 1.5, 8.0)], a chelated hydroxy proton δ 12.80 (s), a methylenedioxy group δ 5.99 (s), methylene protons δ 3.48 (d, 7.0), a trisubstituted olefinic proton δ 5.24 (t, 6.5), and two groups of methyl protons [δ 1.83 (s), δ 1.75 (s)]. The 90 and 135 DEPT spectra indicated resonances for five aromatic methine carbons, ten quaternary carbons, an *α*,*β*-unsaturated ketone carbonyl carbon, a methylenedioxy carbon, a methylene carbon, a methine carbon, and two methyl carbons (Table S1, Fig. S1). The spectroscopic data of isolated Derru (**6**) were compared with those reported (Hastings et al., 2008).

Compound B was identified as isoangustone A (IsoA, **7**) (C_25_H_26_O_6_, light yellow crystals). The ^1^H NMR spectrum showed signals for aromatic protons [δ 8.06 (s), δ 6.26 (s), δ 6.23 (d, 2.0) and δ 6.87 (d, 2.0)], methylene protons [δ 3.40 (d, 7.0), δ 1.78 (s), δ 1.67 (s), δ 3.33 (d, 7.5), δ 1.73 (s), and δ 1.73 (s)], and two trisubstituted olefinic protons [δ 5.20 (m), and δ 5.34 (m)]. The 90 and 135 DEPT spectra indicated resonances for four aromatic methine carbons, twelve quaternary carbons, two methylene carbons, two methine carbons, four methyl carbons, and one *α*,*β*-unsaturated ketone carbonyl carbon (Table S2, Fig. S2). The spectroscopic data of isolated IsoA (**7**) were evaluated in comparison with those reported.(Kiuchi, 1990)

Derru (**6**), IsoA (**7**), and Lup (**2**) were isolated from *D. scandens* leaves as reported previously.(Ito et al., 2020) Derru (**6**), an isoflavone from *Derris robusta* (Roxb. ex DC.) Benth., was identified as a potent Hsp90 inhibitor based on high-throughput screening (Hadden et al., 2007). Derru (**6**) exhibited antiproliferative activity against MCF-7 and SkBr3 breast cancer cell lines (IC_50_ values of 9±0.7 and 12±0.3 μM, respectively).(Hadden et al., 2007) IsoA (**7**) was also isolated from licorice (root of *Glycyrrhiza* sp.) and induces autophagy in colorectal cancer cells.(Tang et al., 2021) IsoA (**7**) exhibited promising antitumor activity *in vivo*, as it significantly inhibited the growth of SW480 human colorectal xenografts in nude mice at 10 mg/kg/day.(Tang et al., 2021) IsoA (**7**) exhibited potent anti-inflammatory activity with an IC_50_ value of 10.8±1.9 μM in inhibiting NO production in LPS-induced RAW 264.7 macrophages.(Wang et al., 2016) Lup (**2**) from *D. scandens* demonstrated anti-inflammatory properties through the inhibition of eicosanoid production.(Laupattarakasem et al., 2004) Lup (**2**) inhibits NO production in LPS-induced RAW 264.7 cells and reduces the expression of pro-inflammatory proteins, including TNF-α, COX-2, and iNOS.(Sae-Foo et al., 2024)

The leaves of *D. scandens* are a valuable source of bioactive compounds. However, a quantitative analytical method for these compounds has not been developed and applied for chemical comparison with stem parts of *D. scandens*.


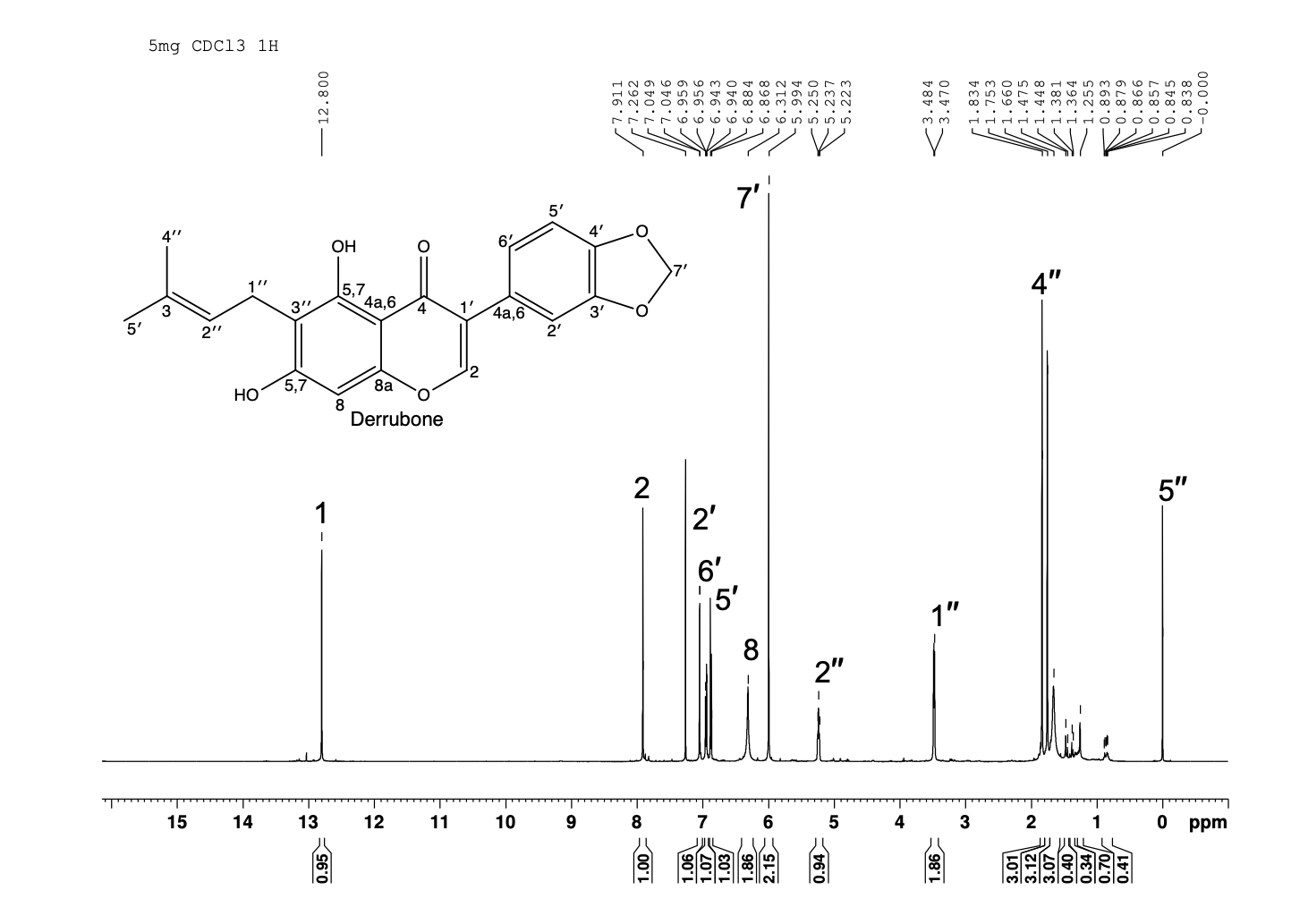


**Fig. S1.** ^1^H-NMR spectrum of derrubone


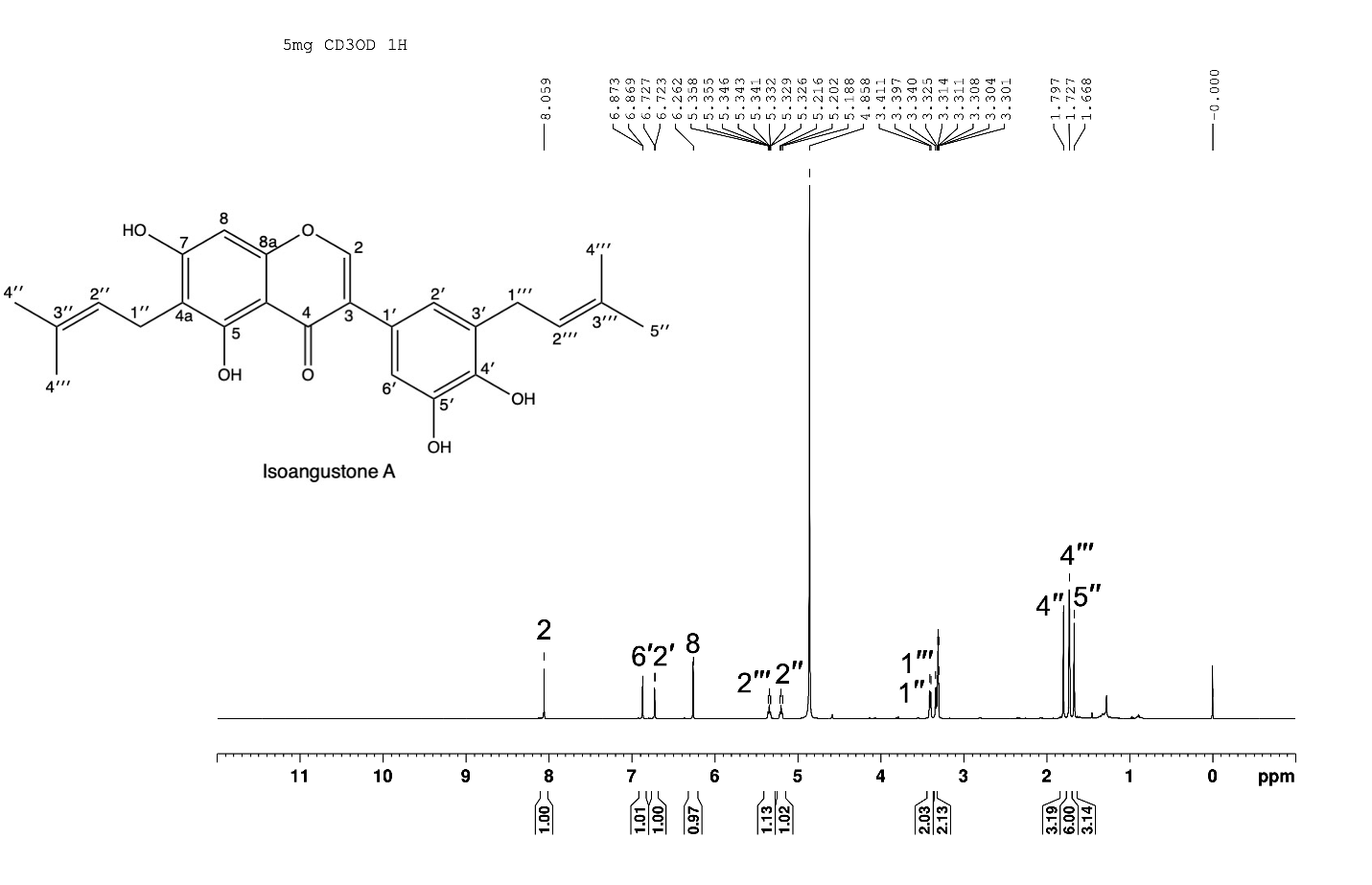


**Fig. S2.** ^1^H-NMR spectrum of isoangustone A

**
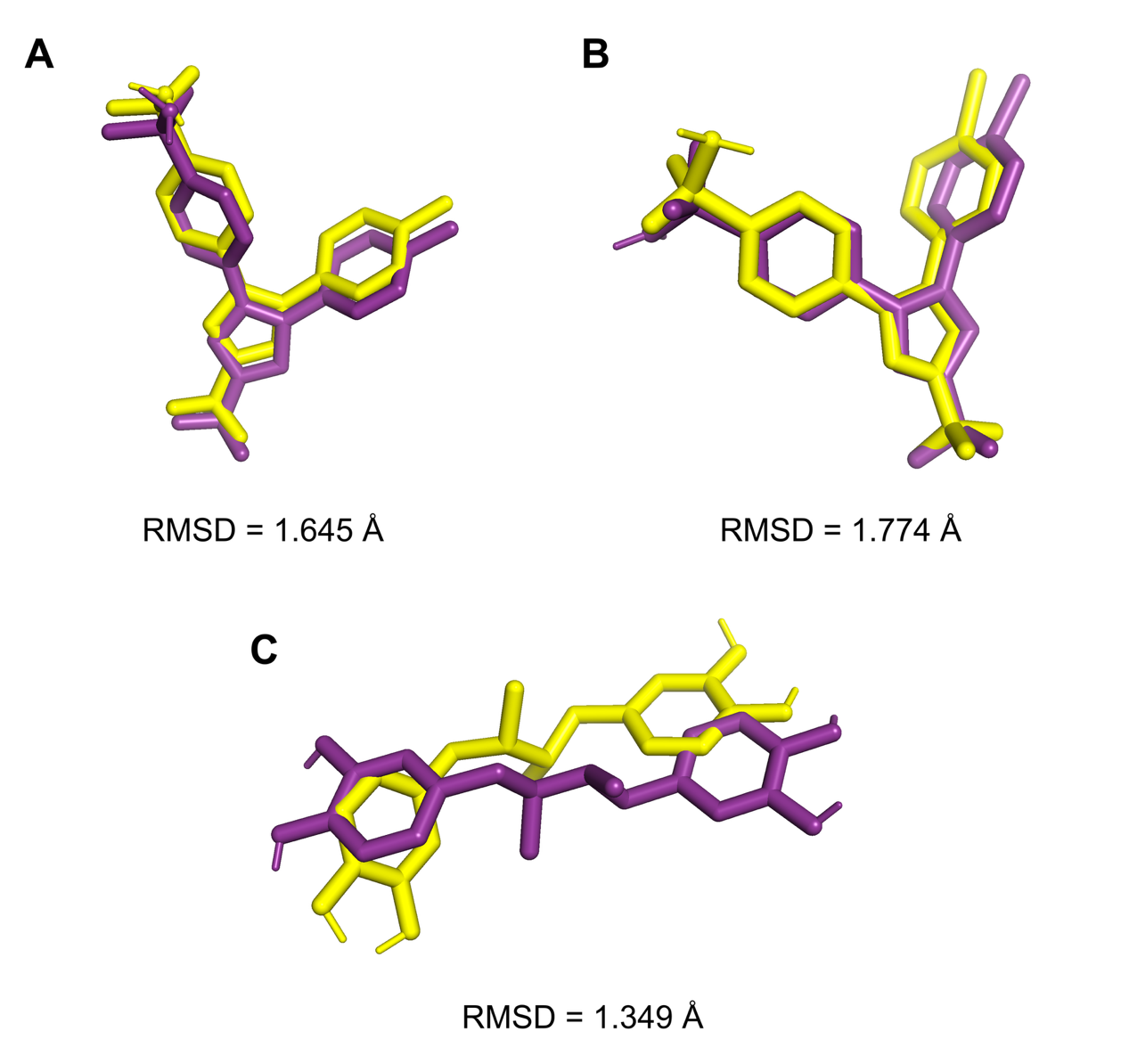
**

**Fig. S3.** (A) Root-mean-square deviation (RMSD) values between redocked and
co-crystallized ligands for celecoxib (CEL). (B) 1-Phenylsulfonamide-3-Trifluoromethyl-5-Parabromophenylpyrazole (S58) and (C) nordihydroguaiaretic acid (30Z) within their respective protein crystal structures. Yellow structures represent redocked ligands, while purple structures represent the co-crystallized ligands.

**
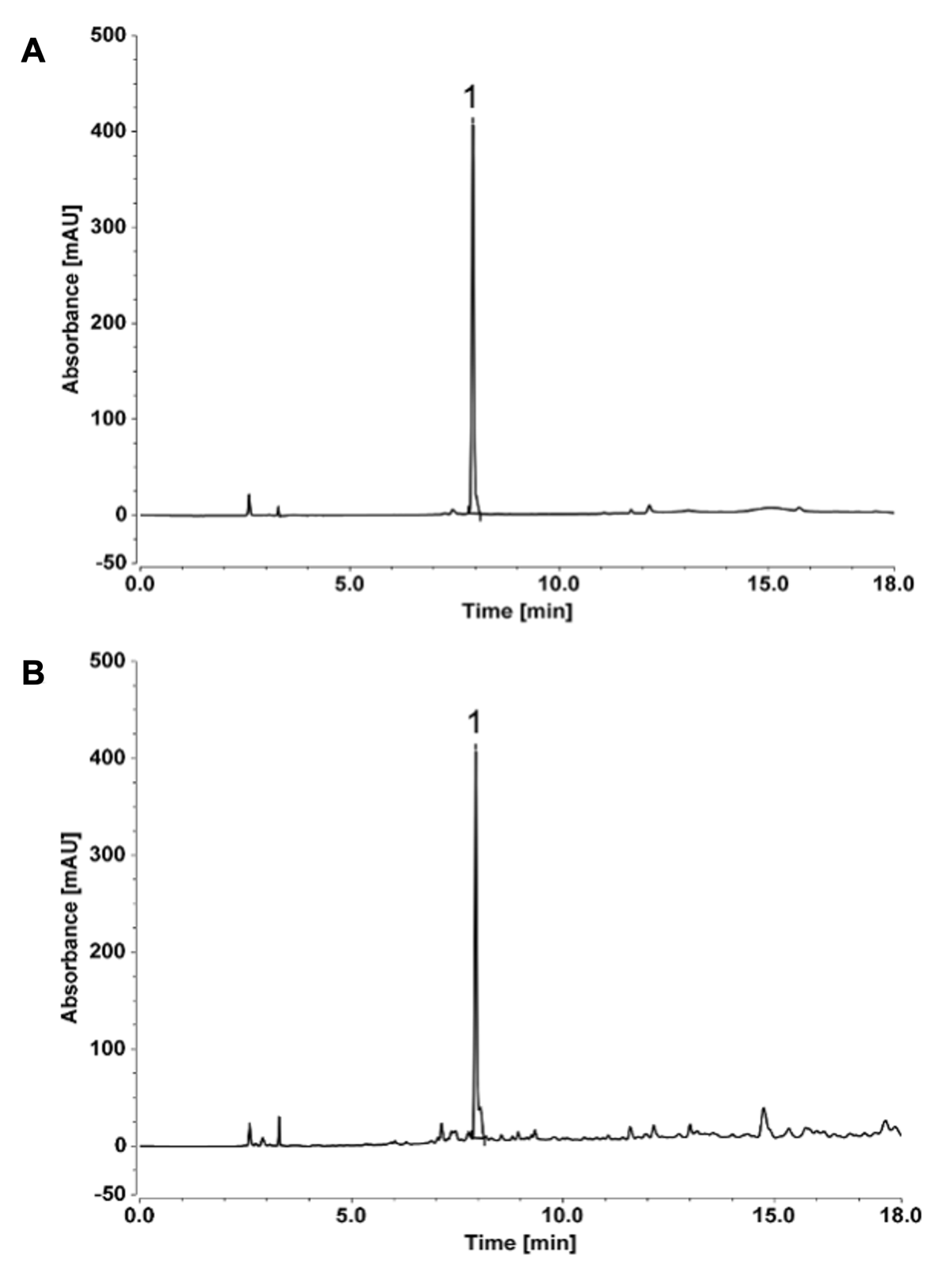
**

**Fig. S4.** HPLC chromatograms of (1) genistein-7-*O*-[α-rhamnopyranosyl-(1→6)]-β-glucopyranoside (GTG, **4**) at 260 nm; A and B are reference standards of GTG (**4**) (200 μg/mL) and the extract of *D. scandens* leaves (3.33 mg/mL), respectively.

**
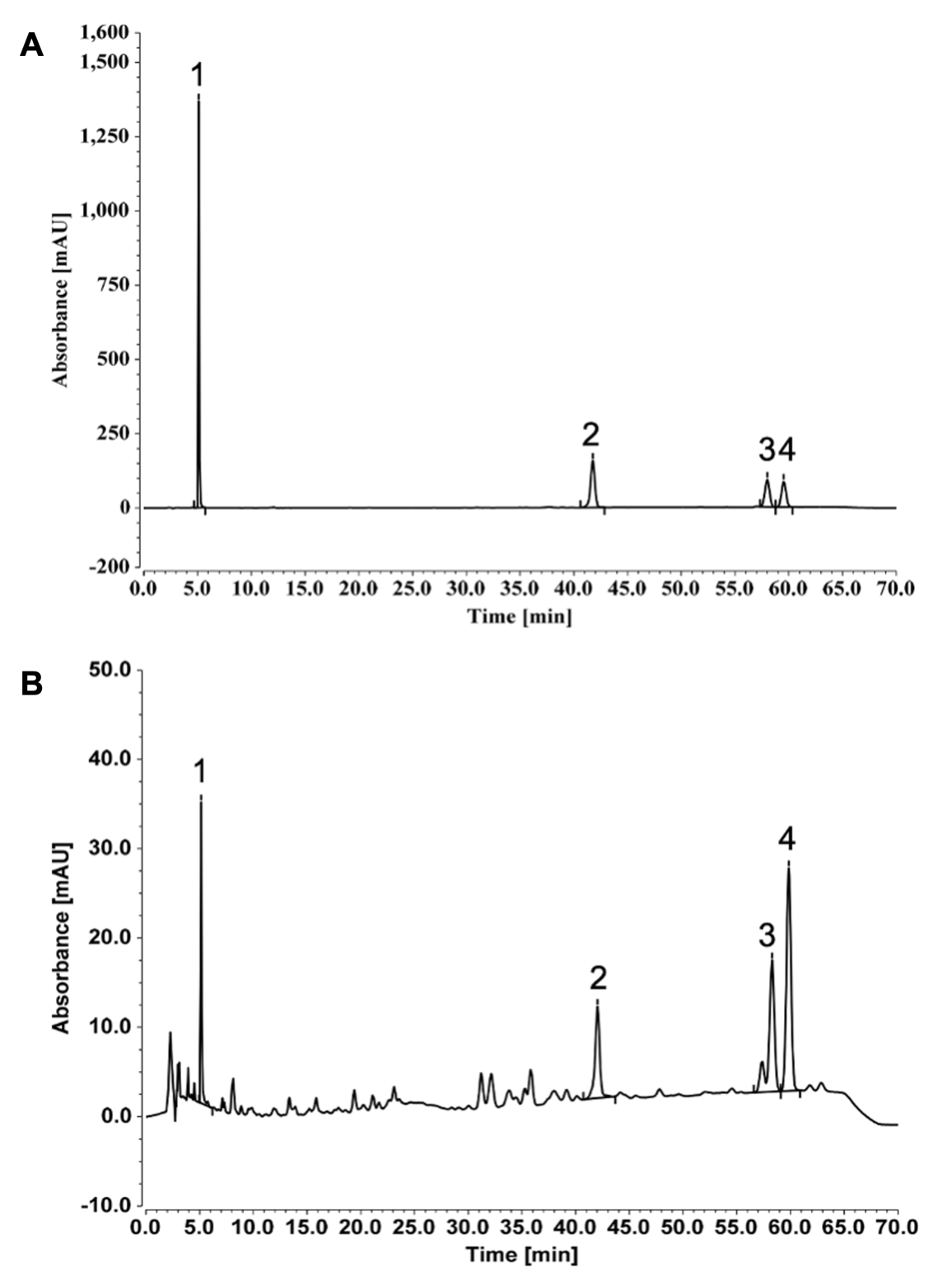
**

**Fig. S5.** HPLC chromatograms of reference compounds including (1) genistein (Gen, **5**), (2) derrisisoflavone A (DerA, **1**), (3) lupalbigenin (Lup, **2**), and (4) 6,8-diprenylgenistein (Dip, **3**); A and B are reference isoflavone derivatives (200 μg/mL), and *D. scandens* stem extracts obtained with 68% ethanol (1.0 mg/mL), respectively.

**Fig. S6.** Effect of the metabolites of *D. scandens* leaves and stems on cell viability in LPS-induced RAW264.7 macrophage cells: (A) metabolites from the leaves and (B) metabolites from the stems (both the leaves and stems contain lupalbigenin (Lup, **2**). Derrubone (Derru, **6**), isoangustone A (IsoA, **7**), lupalbigenin (Lup, **2**), genistein (Gen, **5**), genistein-7-*O*-[α-rhamnopyranosyl-(1→6)]-β-glucopyranoside (GTG, **4**), derrisisoflavone A (DerA, **1**), and 6,8-diprenylgenistein (Dip, **3**) were characterized from the leaves and stems as indicated in the text.

**Fig. S7.** Effects of *D. scandens* leaf and stem extracts on cell viability in lipopolysaccharide-induced RAW264.7 macrophage cells. (A) Leaf material extracted with water and 25–98% ethanol, (B) Leaf extracts from different geographic sources (Lf1–Lf3), and (C) Stems extracted with water and 25–98% ethanol, and (D) Extracts of the stems from different geographic sources (St1–St4).


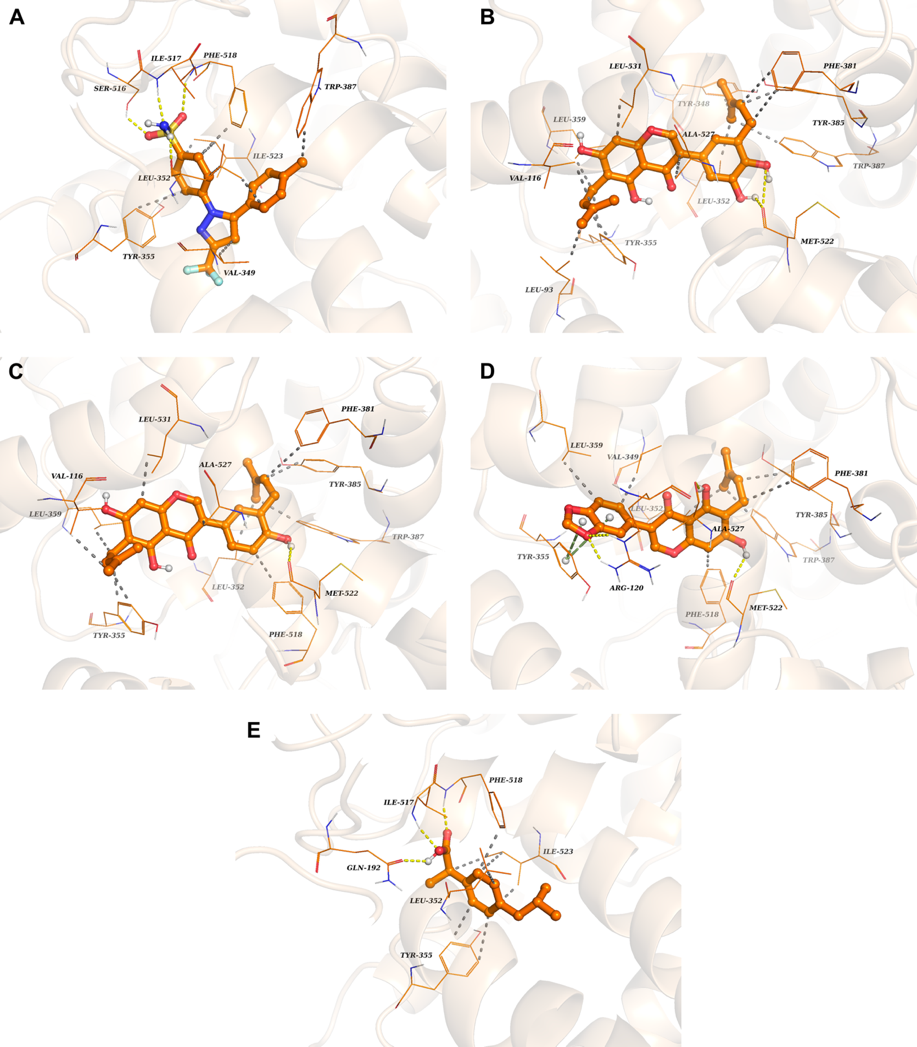


**Fig. S8.** Interactions between ligands and amino acid residues in the active site of COX-1 (PDB ID: 3KK6). (A) The ligands include CEL, the co-crystallized ligand, (B) isoangustone A (IsoA, **7**), (C) lupalbigenin (Lup, **2**), (D) derrubone (Derru, **6**), and (E) ibuprofen. Ligands are shown in ball-and-stick models, with heteroatoms colored as follows: carbon (C) in orange, oxygen (O) in red, nitrogen (N) in blue, sulfur (S) in yellow, fluorine (F) in cyan, and hydrogen (H) in white. Amino acid residues are depicted as line models with heteroatoms labeled according to the same color scheme. The protein backbone is shown as a wheat-colored ribbon. Yellow dashed lines indicate hydrogen bonds, grey dashed lines indicate hydrophobic interactions, and green dashed lines represent π−π stacking interactions between ligand atoms and amino acid residues.


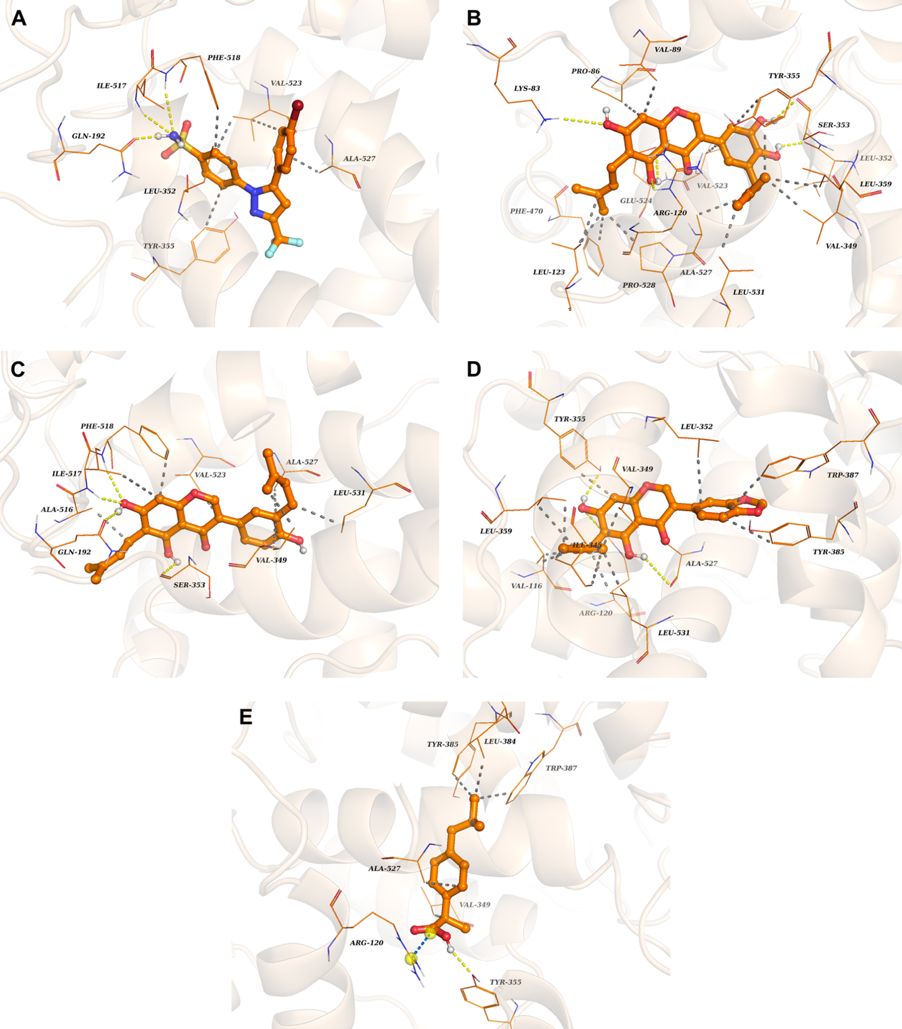


**Fig. S9.** Interactions between ligands and amino acid residues in the active site of COX-2 (PDB ID: 1CX2). (A) The ligands include S58, the co-crystallized ligand, (B) isoangustone A (IsoA, **7**), (C) lupalbigenin (Lup, **2**), (D) derrubone (Derru, **6**), and (E) ibuprofen. Ligands are shown in ball-and-stick models, with heteroatoms colored as follows: carbon (C) in orange, oxygen (O) in red, nitrogen (N) in blue, sulfur (S) in yellow, fluorine (F) in cyan, bromine (Br) in ruby red, and hydrogen (H) in white. Amino acid residues are depicted as line models with heteroatoms labeled according to the same color scheme. The protein backbone is shown as a wheat-colored ribbon. Yellow dashed lines indicate hydrogen bonds, grey dashed lines indicate hydrophobic interactions, and blue dashed lines indicate salt bridge interactions between ligand atoms and amino acid residues.


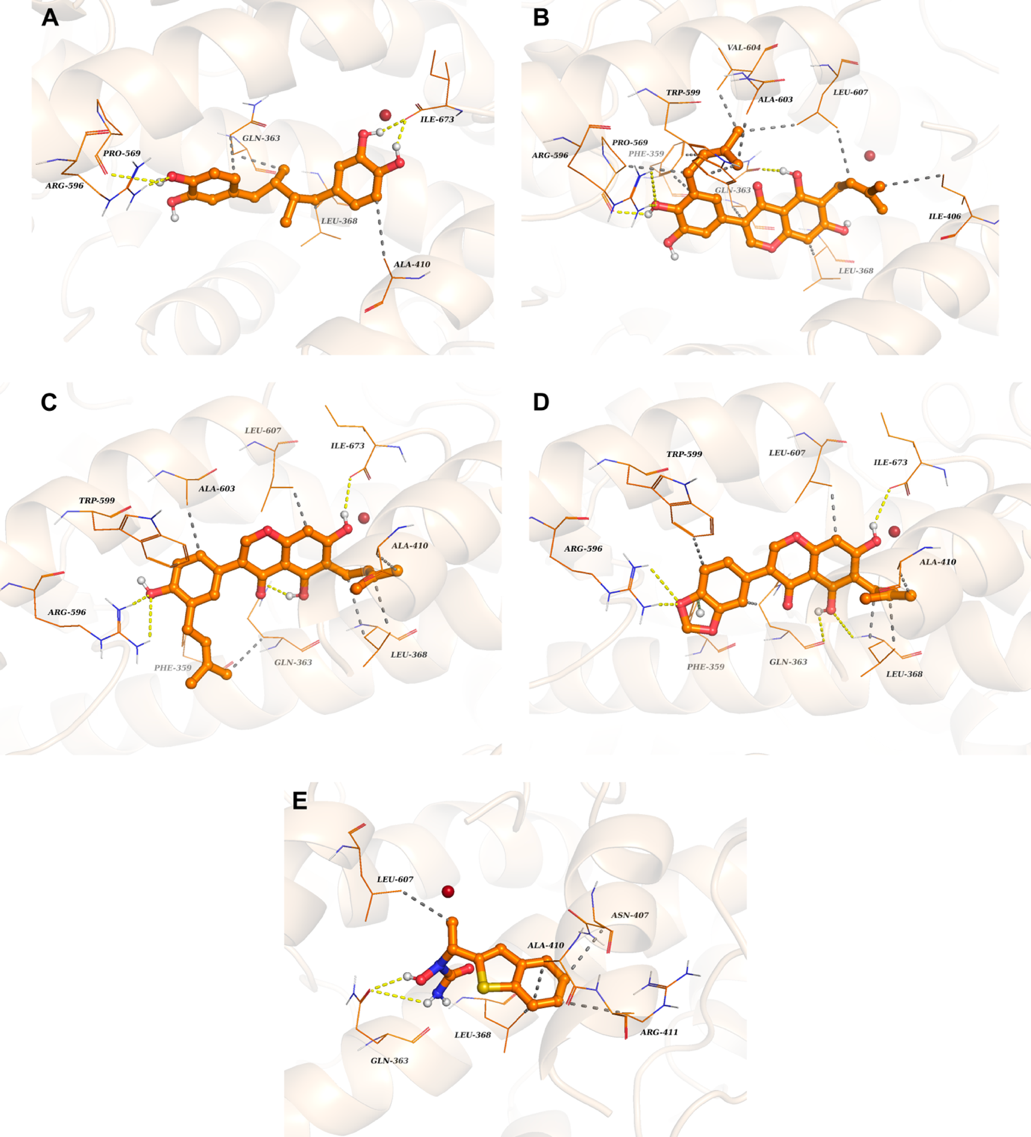


**Fig. S10.** Interactions between ligands and amino acid residues in the active site of 5-LOX (PDB ID: 6N2W). (A) The ligands include 30Z, the co-crystallized ligand, (B) isoangustone A (IsoA, **7**), (C) lupalbigenin (Lup, **2**), (D) derrubone (Derru, **6**), and (E) zileuton. Ligands are shown in ball-and-stick models, with heteroatoms colored as follows: carbon (C) in orange, oxygen (O) in red, nitrogen (N) in blue, sulfur (S) in yellow, and hydrogen (H) in white. Amino acid residues are depicted as line models with heteroatoms labeled according to the same color scheme. The protein backbone is shown as a wheat-colored ribbon. Yellow dashed lines indicate hydrogen bonds, grey dashed lines indicate hydrophobic interactions, and green dashed lines represent π−π stacking interactions between ligand atoms and amino acid residues. The catalytic iron (Fe) is shown as a ruby-red sphere

# **Supplementary Tables**

**Table S1.  ^1^H- and ^13^C-NMR spectral data for derrubone**

| **Position** | ***δ*_H_, mult. (*J*, Hz)** | ***δ*_C_, type** |
| --- | --- | --- |
| 2 | 7.91 (s) | 152.81, CH |
| 3 |  | 123.40, C |
| 4 |  | 181.09, C |
| 4a |  | 106.20, C |
| 5 |  | 160.79^*^, C |
| 5-OH | 12.80 (s) |  |
| 6 |  | 105.30, C |
| 7 |  | 160.68^*^, C |
| 8 | 6.31 (brs) | 99.71, CH |
| 8a |  | 155.03, C |
| 1′ |  | 124.43, C |
| 2′ | 6.88 (d, 8.0) | 108.52, CH |
| 3′ |  | 147.87, C |
| 4′ |  | 147.81, C |
| 5′ | 6.94 (dd, 1.5, 8.0) | 122.46, CH |
| 6′ | 7.05 (d, 1.5) | 109.64, CH |
| 7′ | 5.99 (s) | 101.27, CH_2_ |
| 1″ | 3.48 (d, 7.0) | 21.59, CH_2_ |
| 2″ | 5.24 (t, 6.5) | 121.09, CH |
| 3″ |  | 135.02, C |
| 4″ | 1.83 (s) | 17.91, CH_3_ |
| 5″ | 1.75 (s) | 25.80, CH_3_ |
| 1′′′ |  |  |
| 2′′′ |  |  |
| 3′′′ |  |  |
| 4′′′ |  |  |
| 5′′′ |  |  |

Recorded at 500 and 125 MHz for ^1^H and ^13^C in CDCl_3_, respectively.

^*^interchangeable

**Table S2. ^1^H- and ^13^C-NMR spectra data for compound B (isoangustone A)**

| **Position** | ***δ*_H_, mult. (*J*, Hz)** | ***δ*_C_, type** |
| --- | --- | --- |
| 2 | 8.06 (s) | 154.81, CH |
| 3 |  | 124.82, C |
| 4 |  | 182.71, C |
| 4a |  | 106.29, C |
| 5 |  | 161.45, C |
| 5-OH |  |  |
| 6 |  | 107.94, C |
| 7 |  | 163.27, C |
| 8 | 6.26 (s) | 99.55, CH |
| 8a |  | 156.84, C |
| 1′ |  | 123.10, C |
| 2′ | 6.72 (d, 2.0) | 122.26, CH |
| 3′ |  | 129.77, C |
| 4′ |  | 144.66, C |
| 5′ |  | 145.88, C |
| 6′ | 6.87 (d, 2.0) | 114.84, CH |
| 7′ |  |  |
| 1″ | 3.40 (d, 7.0) | 22.31, CH_2_ |
| 2″ | 5.17 (m) | 123.44, CH |
| 3″ |  | 132.47, C |
| 4″ | 1.80 (s) | 17.93, CH_3_ |
| 5″ | 1.67 (s) | 25.98, CH_3_ |
| 1′′′ | 3.33 (d, 7.5) | 29.36, CH_2_ |
| 2′′′ | 5.34 (m) | 124.03, CH |
| 3′′′ |  | 132.92, C |
| 4′′′ | 1.73 (s) | 25.94, CH_3_ |
| 4′′′ | 1.73 (s) | 25.94, CH_3_ |

Recorded at 500 and 125 MHz for ^1^H and ^13^C in CD_3_OD, respectively.

**Table S3.** Grid box sizes and center coordinates for cyclooxygenase-1 (COX-1), cyclooxygenase-2 (COX-2), and 5-lipoxygenase (5-LOX)

| **Protein−ligand crystal structure** | **Grid box size**  **(x, y, z)** | **Grid box center coordinates** | | | **Grid spacing** | **References** |
| --- | --- | --- | --- | --- | --- | --- |
|  |  | x | y | z |  |  |
| COX-1−CEL  (PDB ID: 3KK6) | 60 × 60 × 60 | −32.501 | 43.578 | −6.333 | 0.375 | **(Saghali et al., 2023)** |
| COX-2−S58  (PDB ID: 1CX2) | 60 × 60 × 60 | 25.862 | 21.902 | 16.940 | 0.375 | **(Saghali et al., 2023; Silva et al., 2015)** |
| 5-LOX−30Z  (PDB ID: 6N2W) | 50 × 50 × 50 | 35.820 | 65.513 | 38.411 | 0.375 | **(Khadri et al., 2024)** |

CEL: Celecoxib; S58: 1-Phenylsulfonamide-3-Trifluoromethyl-5-Parabromophenylpyrazole; and 30Z: Nordihydroguaiaretic acid

**Table S4. Sensitivity, calibration range, and correlation coefficients of the HPLC systems**

| **Analytes** | **Analytical parameters** | | | | |
| --- | --- | --- | --- | --- | --- |
|  | **Calibration range**  **(μg/mL)** | **Equation** | **(R^2^)** | **LOD**  **(μg/mL)** | **LOQ**  **(μg/mL)** |
| HPLC system 1 | | | | | |
| Derru | 0.78–200 | y = 0.7928x + 1.1018 | 0.9995 | 0.20 | 0.61 |
| IsoA | 0.39–200 | y = 0.4629x + 0.5755 | 0.9994 | 0.08 | 0.24 |
| Lup | 0.78–200 | y = 0.3267x + 0.4835 | 0.9993 | 0.23 | 0.71 |
| HPLC system 2 | | | | | |
| GTG | 1.56–100 | y = 4.327x + 1.956 | 0.9988 | 0.07 | 0.22 |
| HPLC system 3 | | | | | |
| Gen | 1.56–200 | y = 6.8689x + 6.1128 | 0.9998 | 0.35 | 1.05 |
| DerA | 0.78–200 | y = 0.7927x + 0.7397 | 0.9999 | 0.25 | 0.76 |
| Dip | 1.56–200 | y = 0.5842x + 0.4731 | 0.9998 | 0.30 | 0.90 |
| Lup | 0.78–200 | y = 0.4945x + 0.2431 | 0.9997 | 0.13 | 0.41 |

**Table S5.** **Accuracy and precision of HPLC system-1**

| **Analytes** | **Spiked concentration**  **(µg/mL)** | **Measured concentration (µg/mL)** | **Recovery (%)** | **CV (%)** |
| --- | --- | --- | --- | --- |
|  |  |  |  |  |
| Derru | 25 | 35.3±2.7 | 96.0 | 0.43 |
|  | 50 | 57.7±5.8 | 99.2 | 0.47 |
| IsoA | 25 | 73.5±8.4 | 101 | 0.58 |
|  | 50 | 107±17 | 102 | 0.56 |
| Lup | 25 | 22.3±3.9 | 96.2 | 0.14 |
|  | 50 | 41.7±12.7 | 99.4 | 0.44 |

**Table S6.** **Accuracy and precision of HPLC system-2**

| **Analyte** | **Spiked concentration**  **(µg/mL)** | **Measured concentration (µg/mL)** | **Recovery (%)** | **CV (%)** |
| --- | --- | --- | --- | --- |
| GTG | 10  20  40  60 | 9.78±0.11  20.4±0.1  40.9±0.6  61.8±0.8 | 97.8  102  102  103 | 1.08  0.62  1.43  1.34 |

**Table S7.** **Accuracy and precision of HPLC systems-3**

| **Analytes** | **Spiked concentration**  **(µg/mL)** | **Measured concentration (µg/mL)** | **Recovery (%)** | **CV (%)** |
| --- | --- | --- | --- | --- |
| Gen | 6.25 | 5.46±1.47 | 104 | 0.69 |
|  | 25 | 24.5±7.7 | 97.2 | 0.17 |
|  | 50 | 34.5±13.2 | 95.7 | 0.94 |
| DerA | 6.25 | 12.7±1.2 | 103 | 0.40 |
|  | 25 | 32.8±0.4 | 104 | 1.03 |
|  | 50 | 59.5±6.5 | 95.9 | 0.44 |
| Dip | 6.25 | 22.5±0.8 | 102 | 0.49 |
|  | 25 | 44.4±1.1 | 102 | 0.39 |
|  | 50 | 69.7±3.9 | 102 | 1.07 |
| Lup | 6.25 | 36.0±0.7 | 102 | 0.79 |
|  | 25 | 59.1±1.7 | 93.6 | 0.18 |
|  | 50 | 84.1±4.11 | 103 | 1.15 |

**Table S8.** Effects of the metabolites of *D. scandens* leaves and stems on the inhibition of NO secretion in LPS-induced RAW264.7 macrophage cells: chemical constituents from the leaves and chemical constituents from the stems (both leaves and stems contain Lup (**2**)). The metabolites are derrubone (Derru, **6**), isoangustone A (IsoA, **7**), lupalbigenin (Lup, **2**), genistein (Gen, **5**), genistein-7-*O*-[α-rhamnopyranosyl-(1→6)]-β-glucopyranoside (GTG, **4**), derrisisoflavone A (DerA, **1**), and 6,8-diprenylgenistein (Dip, **3**). Data are presented as mean ± SD (*n* = 3).

| **Concentration (μg/mL)** | **Inhibition of nitric oxide (%)** | | | | | | |
| --- | --- | --- | --- | --- | --- | --- | --- |
|  | **Derru (6)** | **IsoA (7)** | **Lup (2)** | **Gen (5)** | **GTG (4)** | **DerA (1)** | **Dip (3)** |
| 6.25 | No inhibition | No inhibition | No inhibition | 18.9±3.9 | 10.3±3.5 | 11.1±4.4 | 11.0±2.1 |
| 12.5 | 11.9±6.1 | 0.50±6.01 | 1.64±2.36 | 27.1±1.8 | 9.00±3.21 | 17.3±1.9 | 17.2±3.8 |
| 25 | 31.0±3.0 | 18.0±3.3 | 9.17±3.22 | 36.9±2.8 | 7.61±3.82 | 34.3±3.2 | 23.3±3.0 |
| 50 | 52.5±3.1 | 42.4±2.6 | 28.1±2.2 | 46.0±2.3 | 3.26±2.95 | ND | ND |

^a^ No inhibition: no detectable inhibitory effect on nitric oxide production at the tested concentration

^b^ ND: not determined due to cell viability <80%, therefore nitric oxide inhibition was not evaluated at this concentration

**Table S9.** Inhibition of NO production in LPS-stimulated RAW 264.7 macrophages by *D. scandens* extracts. Leaf extracts (Lf1) obtained using different ethanol concentrations. Leaf extracts from different locations obtained using 98% ethanol. Stem extracts (St1) obtained using different ethanol concentrations. Stem extracts from different locations obtained using 98% ethanol. Data are presented as mean ± SD (*n* = 3).

| **Concentration (μg/mL)** | **Inhibition of nitric oxide (%)** | | | | | | | | |
| --- | --- | --- | --- | --- | --- | --- | --- | --- | --- |
|  | **Lf1-75E** | **Lf1-98E** | **Lf2-98E** | **Lf3-98E** | **St1-75E** | **St1-98E** | **St2-98E** | **St3-98E** | **St4-98E** |
| 12.5 | No inhibition | No inhibition | No inhibition | No inhibition | 4.93±3.84 | 12.1±2.9 | 16.9±2.3 | 4.21±4.19 | 16.1±2.6 |
| 25 | 5.60±5.07 | 4.60±4.76 | 1.32±3.70 | 3.15±3.42 | 9.14±4.37 | 21.5±2.3 | 30.1±1.2 | 6.35±2.59 | 28.1±3.3 |
| 50 | 39.2±5.0 | 6.59±3.09 | 22.3±3.7 | 16.1±2.5 | 11.4±3.9 | 39.8±2.4 | 59.6±3.4 | 9.44±3.21 | 52.2±2.2 |
| 100 | 77.4±1.5 | 11.8±3.8 | 66.0±3.1 | 52.4±2.7 | 23.8±2.9 | 67.6±0.7 | ND | 26.0±3.1 | ND |

^a^ No inhibition: no detectable inhibitory effect on nitric oxide production at the tested concentration

^b^ ND: not determined due to cell viability <80%, therefore nitric oxide inhibition was not evaluated at this concentration

**Table S10.** Binding energies and key interacting amino acid residues of isolated compounds with targeted anti-inflammatory proteins

| **Target** | **Compounds** | **Binding energy (kcal/mol)** | **Hydrogen bonds** | **Hydrophobic interactions** | **π−π stacking interactions** | **Salt bridge interactions** | **Inhibition constant**  **(*K*_i_)** |
| --- | --- | --- | --- | --- | --- | --- | --- |
|  |  |  | **Residues** | **Residues** | **Residues** | **Residues** |  |
| COX-1  (PDB ID: 3KK6) | 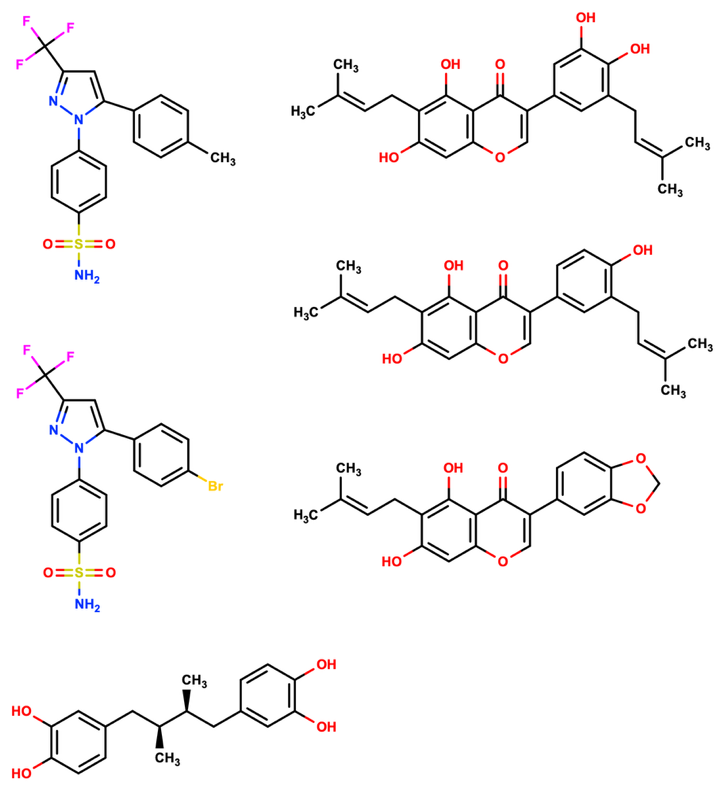  CEL (co-crystallized ligand) | −12.06 | LEU352, SER516, ILE517, PHE518 | VAL349, LEU352^a^, TYR355, TRP387, PHE518, ILE523^a^ | - | - | 1.46 nM |
|  | 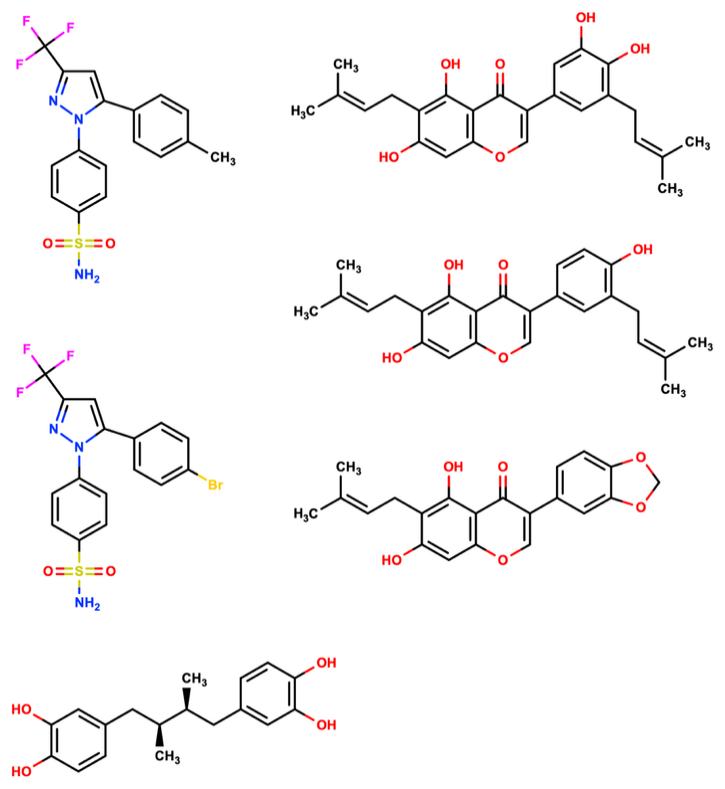  Isoangustone A | −8.06 | MET522^a^ | LEU93, VAL116, TYR348, LEU352, TYR355^a^, LEU359, PHE381^a^, TYR385, TRP387, ALA527, LEU531 | - | - | 1.24 μM |
|  | 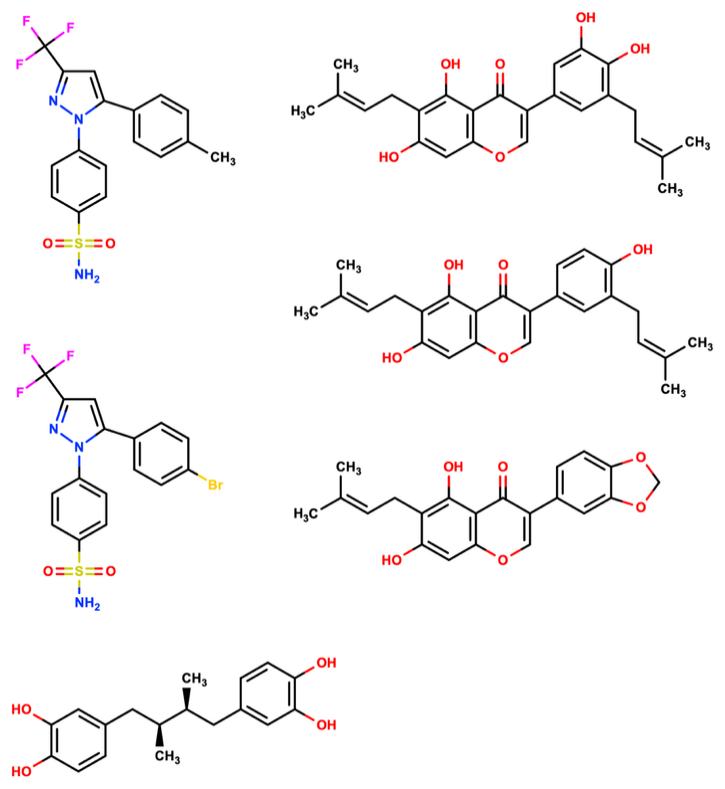  Lupalbigenin | −8.76 | MET522 | VAL116, LEU352, TYR355^a^, LEU359, PHE381, TYR385, TRP387, PHE518, ALA527, LEU531 | - | - | 381.12 nM |
|  | 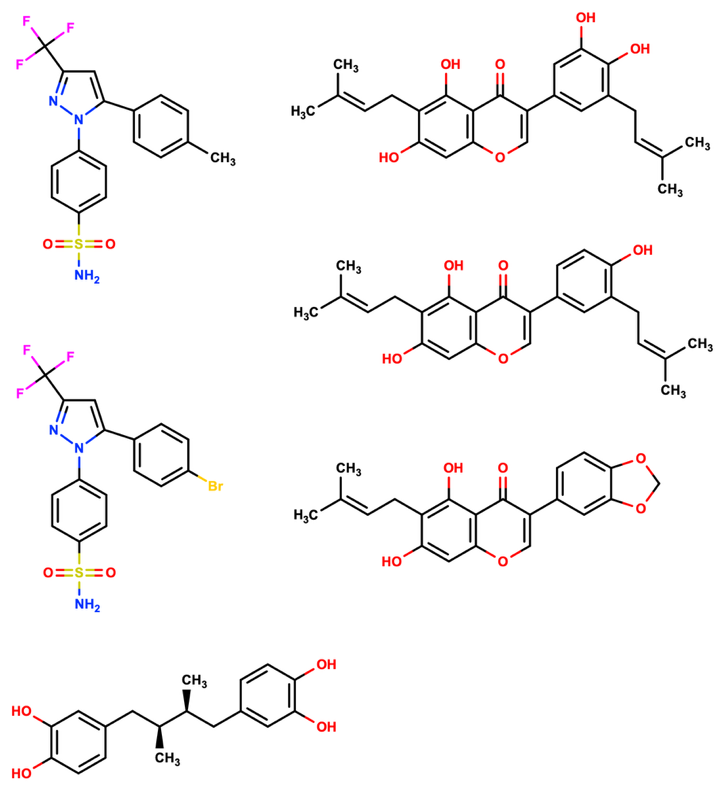  Derrubone | −8.11 | ARG120^a^, MET522, ALA527 | VAL349, LEU352, LEU359, PHE381, TYR385, TRP387, PHE518 | TYR355^a^ | - | 1.14 μM |
|  | 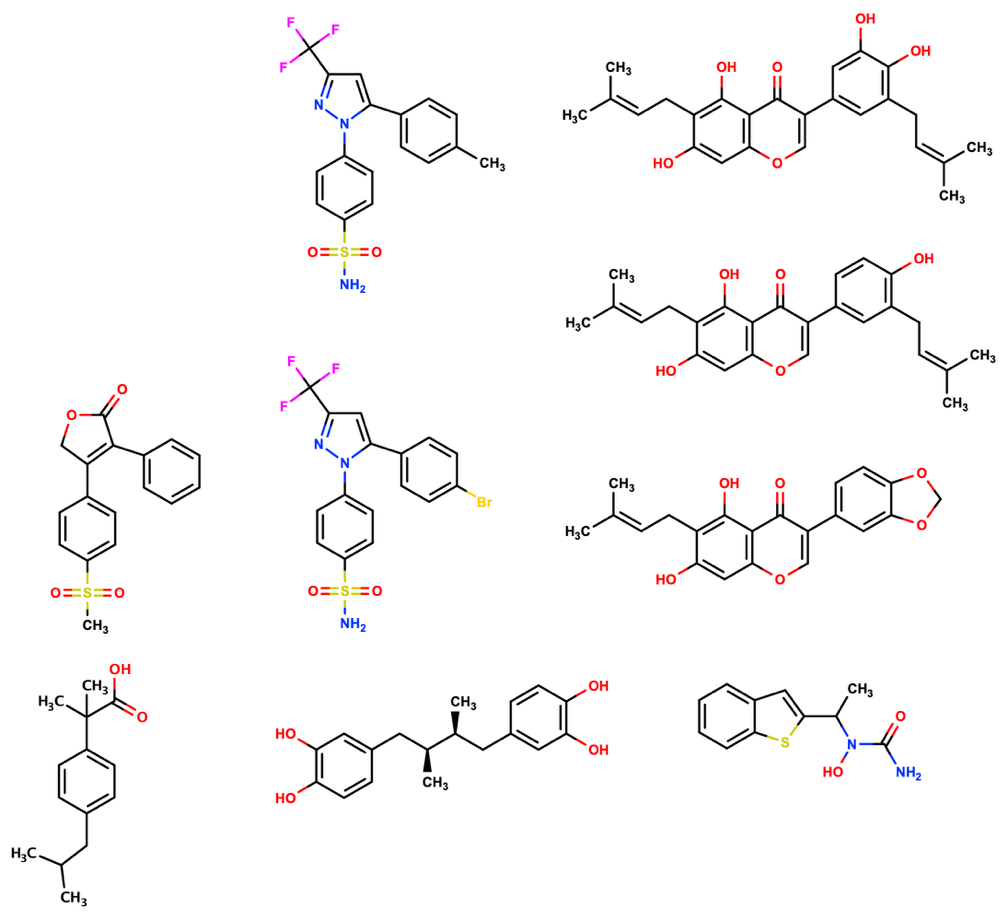  Ibuprofen | −7.80 | GLN192, ILE517, PHE518 | LEU352, TYR355^a^, PHE518, ILE523^b^ | - | - | 1.92 μM |
| COX-2  (PDB ID: 1CX2) | **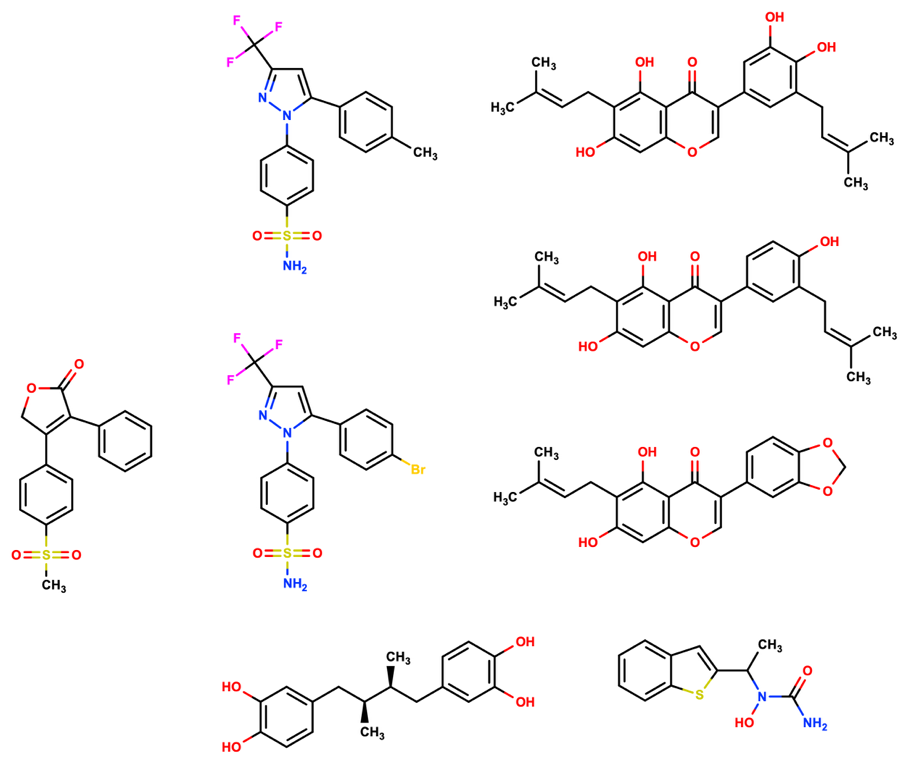**  S58 (co-crystallized ligand) | −11.24 | GLN192, ILE517, PHE518 | LEU352, TYR355, PHE518, VAL523^a^, ALA527 | - | - | 5.79 nM |
|  | 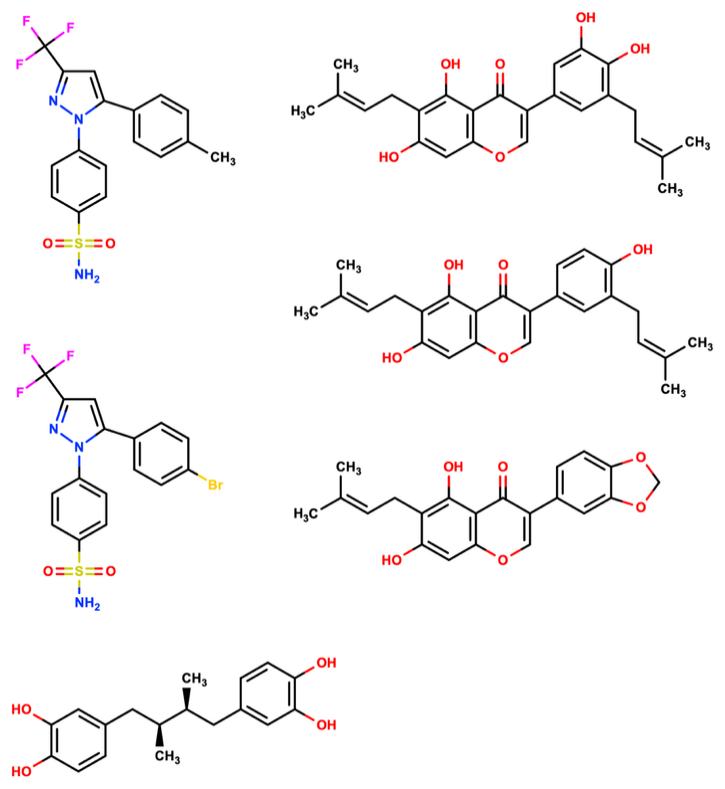Isoangustone A | −5.61 | LYS83, ARG120, LEU352, SER353, GLU524 | PRO86, VAL89, LEU123^a^, VAL349, TYR355^a^, LEU359, PHE470, VAL523, ALA527, PRO528, LEU531 | - | - | 77.18 μM |
|  | 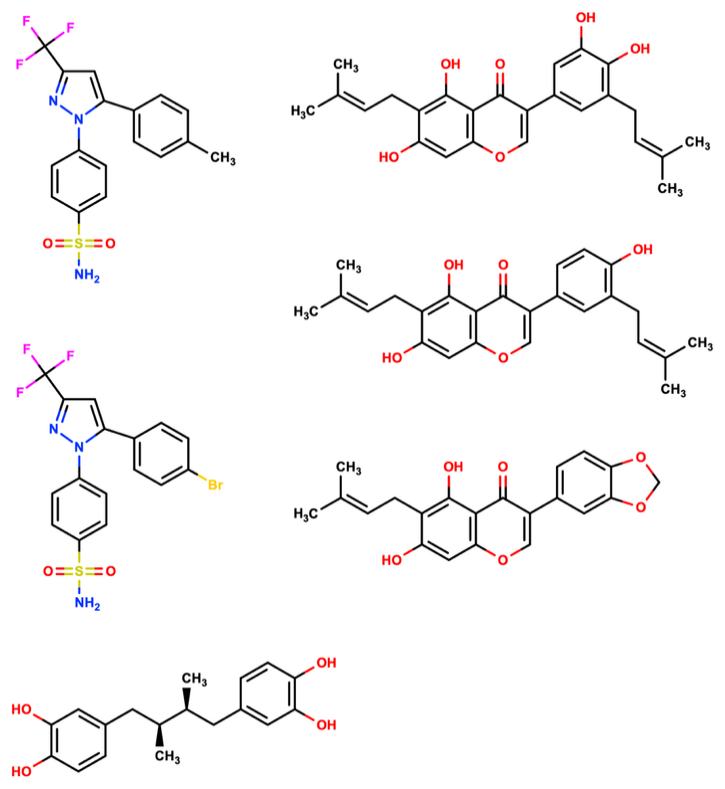Lupalbigenin | −8.98 | GLN192, SER353, ILE517, PHE518 | VAL349^a^, ALA516, ILE517, PHE518, VAL523, ALA527, LEU531 | - | - | 262.77 nM |
|  | 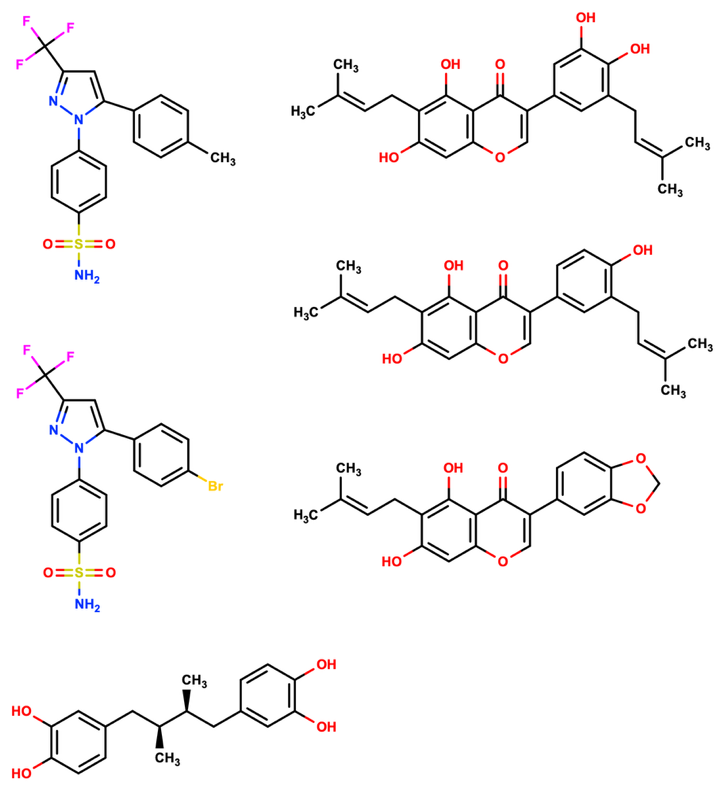  Derrubone | −6.89 | ARG120, TYR355, ALA527 | VAL116^a^, ILE345^a^, VAL349, LEU352, TYR355, LEU359^a^, TYR385, TRP387, LEU531^a^ | - | - | 8.97 μM |
|  | 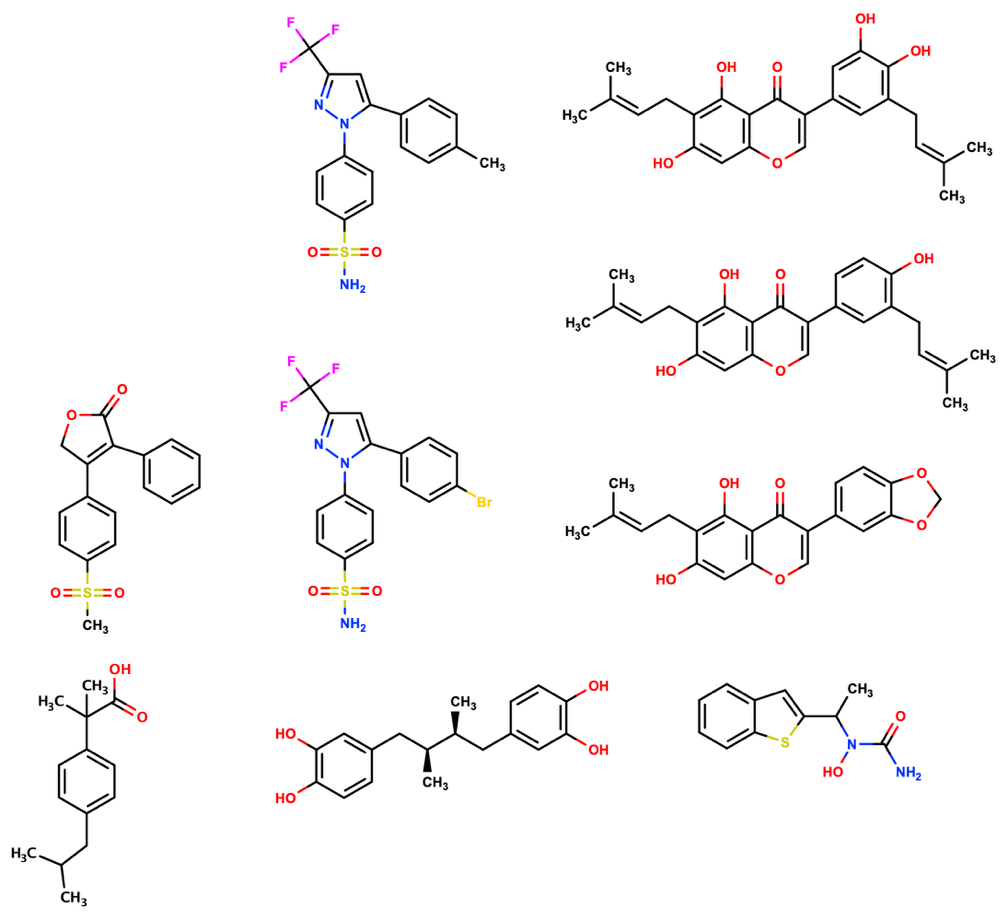  Ibuprofen | −6.36 | TYR355 | VAL349, LEU384, TYR385, TRP387, ALA527 | - | ARG120 | 21.89 μM |
| 5-LOX  (PDB ID: 6N2W) | 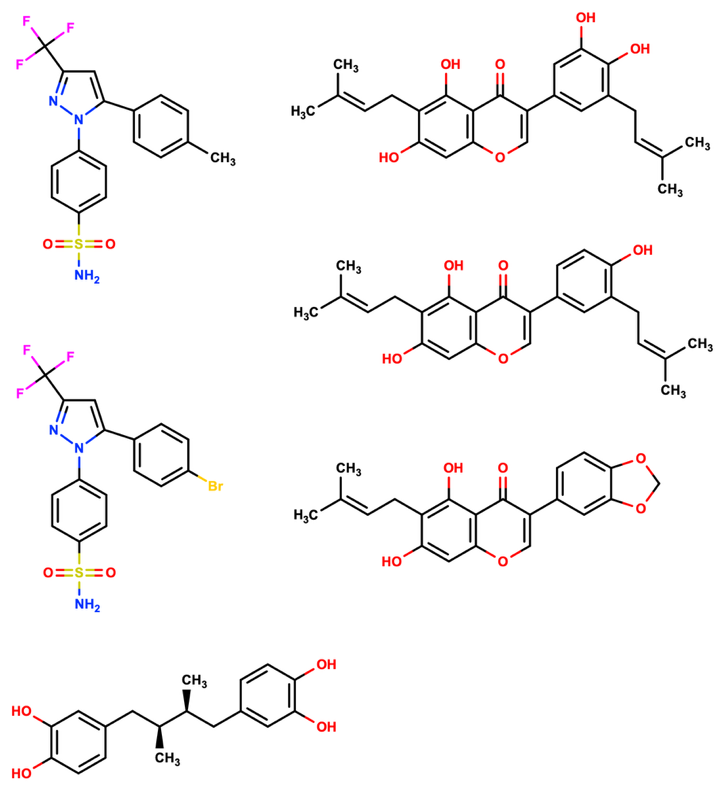  30Z (co-cryatallized ligand) | −5.10 | PRO569, ARG596, ILE673^a^ | GLN363^a^, LEU368, ALA410 | - | - | 182.19 μM |
|  | 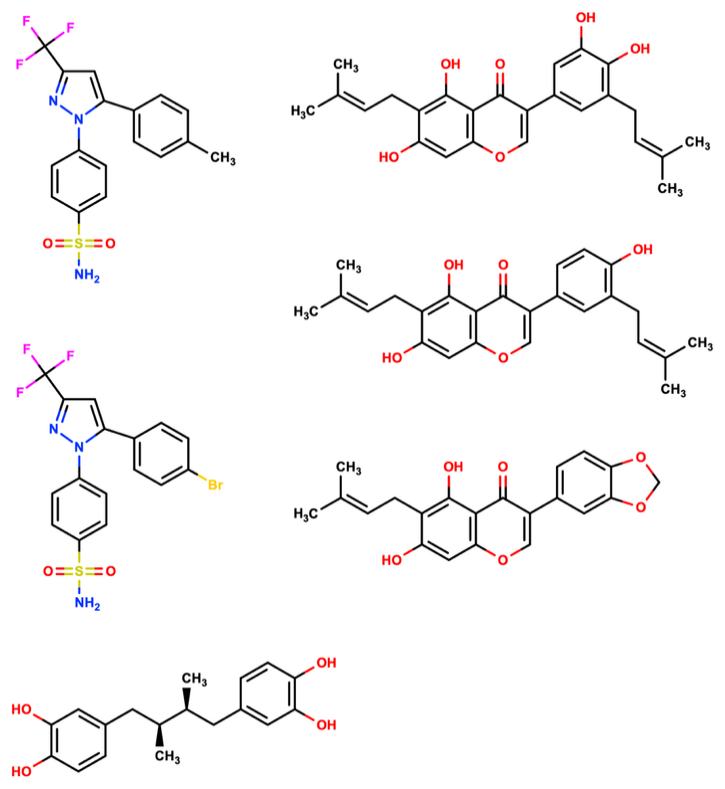  Isoangustone A | −7.45 | GLN363, PRO569, ARG596^a^ | PHE359^a^, GLN363, LEU368, ILE406, PRO569, TRP599, ALA603, VAL604, LEU607^a^ | - | - | 3.48 μM |
|  | 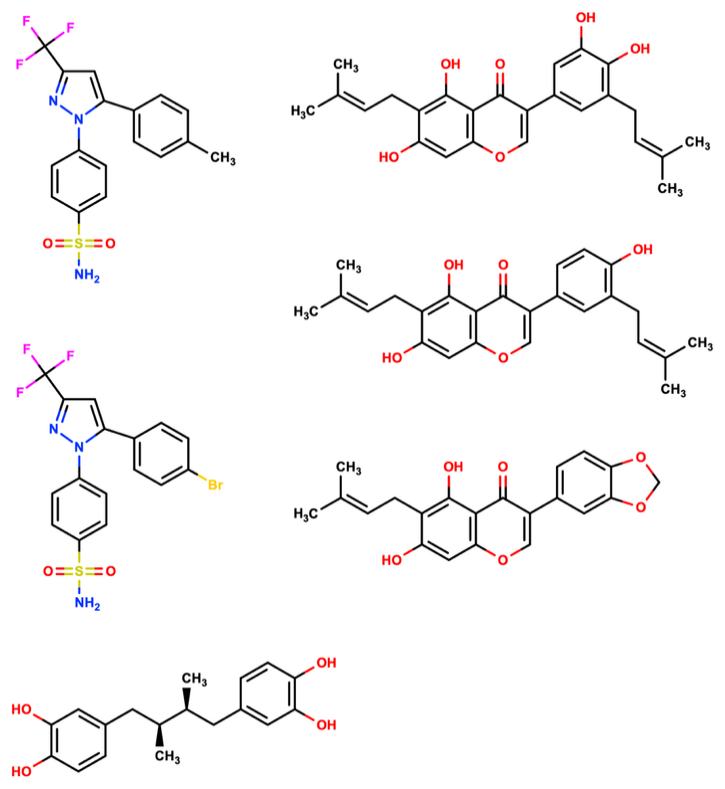  Lupalbigenin | −7.55 | GLN363, ARG596^a^, ILE673 | PHE359^a^, GLN363, LEU368^a^, ALA410, TRP599, ALA603, LEU607 | - | - | 2.91 μM |
|  | 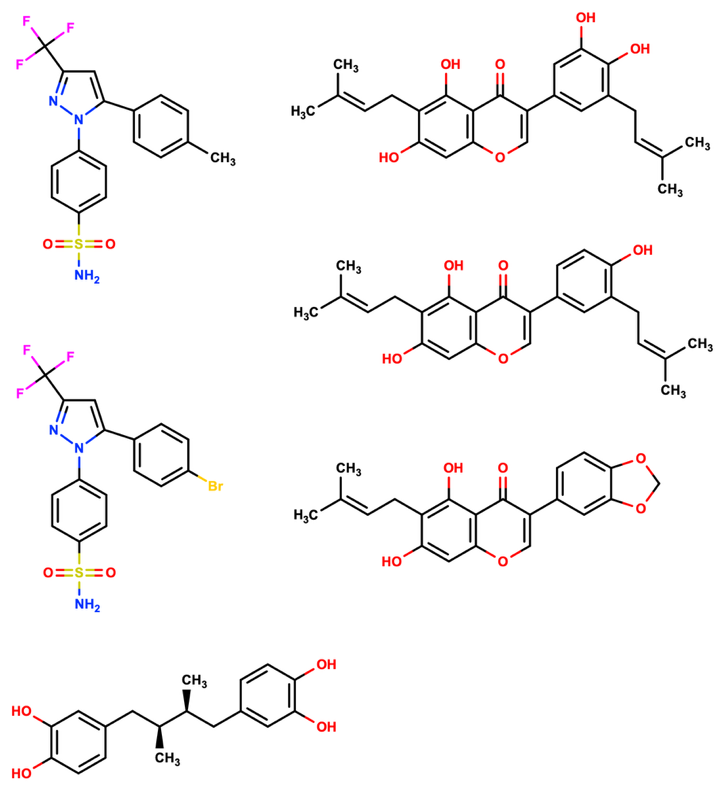  Derrubone | −6.32 | GLN363, LEU368, ARG596^a^, ILE673 | PHE359, GLN363, LEU368^a^, ALA410, TRP599, LEU607 | PHE359 | - | 23.29 μM |
|  | 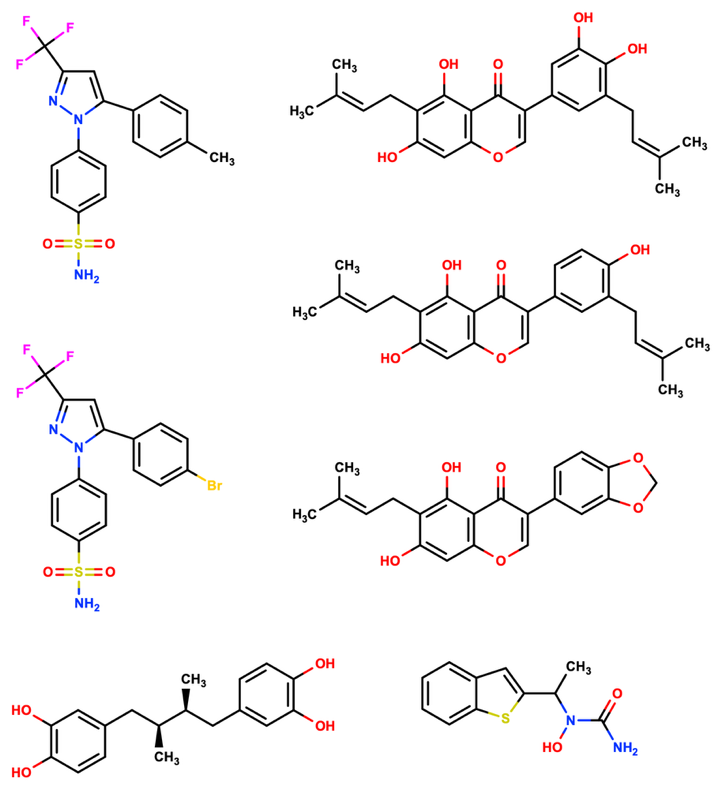  Zileuton (5-LOX inhibitor) | −5.45 | GLN363^a^ | LEU368, ASN407, ALA410, ARG411, LEU607 | - | - | 101.76 μM |

CEL: Celecoxib; S58: 1-Phenylsulfonamide-3-Trifluoromethyl-5-Parabromophenylpyrazole; and 30Z: Nordihydroguaiaretic acid

^a^ Two interacting amino acid residues; ^b^ Three interacting amino acid residues

**References**

Hadden, M. K., Galam, L., Gestwicki, J. E., Matts, R. L., & Blagg, B. S. (2007). Derrubone, an inhibitor of the Hsp90 protein folding machinery. *J. Nat. Prod.*, *70*(12), 2014-2018. <https://doi.org/10.1021/np070190s>

Hastings, J. M., Hadden, M. K., & Blagg, B. S. (2008). Synthesis and evaluation of derrubone and select analogues. *J. Org. Chem.*, *73*(2), 369-373. <https://doi.org/10.1021/jo702366g>

Ito, C., Matsui, T., Miyabe, K., Hasan, C. M., Rashid, M. A., Tokuda, H., & Itoigawa, M. (2020). Three isoflavones from *Derris scandens* (Roxb.) Benth and their cancer chemopreventive activity and *in vitro* antiproliferative effects. *Phytochemistry*, *175*, 112376. <https://doi.org/10.1016/j.phytochem.2020.112376>

Khadri, M. J. N., Ramu, R., Simha, N. A., & Khanum, S. A. (2024). Synthesis, molecular docking, analgesic, anti-inflammatory, and ulcerogenic evaluation of thiophene-pyrazole candidates as COX, 5-LOX, and TNF-α inhibitors. *Inflammopharmacology*, *32*(1), 693-713. <https://doi.org/10.1007/s10787-023-01364-0>

Kiuchi, F., Chen, X., Tsuda, Y.,. (1990). Four new phenolic constituents from licorice (root of *Glycyrrhiza* sp.). *Heterocycles*, *31*, 629–636.

Laupattarakasem, P., Houghton, P. J., & Hoult, J. R. (2004). Anti-inflammatory isoflavonoids from the stems of *Derris scandens*. *Planta Med*, *70*(6), 496-501. <https://doi.org/10.1055/s-2004-827147>

Sae-Foo, W., Yusakul, G., Nualkaew, N., & Putalun, W. (2024). Identification of major bioactive anti-inflammatory compounds of *Derris scandens* stem using RAW 264.7 cells and HPLC-UV analysis. *Planta Med.*, *90*(2), 126-137. <https://doi.org/10.1055/a-2192-2281>

Saghali, M., Lemeski, E. T., Baraghoosh, M. F., Mirzaei, H., Khandoozi, S. R., Erfani-Moghadam, V., Taziki, S., & Soltani, A. (2023). Density functional theory and molecular docking studies on electronic and optical features of poly (lactic acid) interacting with celecoxib. *Chemical Physics Impact*, *7*, 100356. <https://doi.org/https://doi.org/10.1016/j.chphi.2023.100356>

Silva, T., Borges, F., Edraki, N., Alizadeh, M., Miri, R., Saso, L., & Firuzi, O. (2015). Hydroxycinnamic acid as a novel scaffold for the development of cyclooxygenase-2 inhibitors [10.1039/C5RA08692B]. *RSC Advances*, *5*(72), 58902-58911. <https://doi.org/10.1039/C5RA08692B>

Tang, S., Cai, S., Ji, S., Yan, X., Zhang, W., Qiao, X., Zhang, H., Ye, M., & Yu, S. (2021). Isoangustone A induces autophagic cell death in colorectal cancer cells by activating AMPK signaling. *Fitoterapia*, *152*, 104935. <https://doi.org/10.1016/j.fitote.2021.104935>

Wang, S., Wang, H., Liu, Y., Wang, Y., Fan, X., & Cheng, Y. (2016). Rapid discovery and identification of anti-inflammatory constituents from traditional Chinese medicine formula by activity index, LC-MS, and NMR. *Sci. Rep.*, *6*(1), 31000. <https://doi.org/10.1038/srep31000>
